# Model-Based Characterization of the Pharmacokinetics, Target Engagement Biomarkers, and Immunomodulatory Activity of PF-06342674, a Humanized mAb Against IL-7 Receptor-α, in Adults with Type 1 Diabetes

**DOI:** 10.1208/s12248-019-0401-3

**Published:** 2020-01-03

**Authors:** Jason H. Williams, Chandrasekhar Udata, Bishu J. Ganguly, Samantha L. Bucktrout, Tenshang Joh, Megan Shannon, Gilbert Y. Wong, Matteo Levisetti, Pamela D. Garzone, Xu Meng

**Affiliations:** 1 Worldwide Research & Development, Pfizer Inc, 10777 Science Center Dr, CB1/1130, San Diego, California 92121 USA; 2 Pfizer Inc, South San Francisco, California USA; 3 Present Address: Lyell Immunopharma, South San Francisco, California USA; 4 Present Address: Parker Institute for Cancer Immunotherapy, San Francisco, California USA; 5 Present Address: DNAtrix Therapeutics, San Diego, California USA; 6 Present Address: Calibr, a division of Scripps Research, La Jolla, California USA

**Keywords:** autoimmune diabetes, dose response, effector memory, IL-7 receptor, population pharmacokinetic/pharmacodynamics model, target-mediated drug disposition

## Abstract

IL-7 receptor-α (IL-7Rα) blockade has been shown to reverse autoimmune diabetes in the non-obese diabetic mouse by promoting inhibition of effector T cells and consequently altering the balance of regulatory T (T_reg_) and effector memory (T_EM_) cells. PF-06342674 is a humanized monoclonal antibody that binds to and inhibits the function of IL-7Rα. In the current phase 1b study, subjects with type 1 diabetes (T1D) received subcutaneous doses of either placebo or PF-06342674 (1, 3, 8 mg/kg/q2w or 6 mg/kg/q1w) for 10 weeks and were followed up to 18 weeks. Nonlinear mixed effects models were developed to characterize the pharmacokinetics (PK), target engagement biomarkers, and immunomodulatory activity. PF-06342674 was estimated to have 20-fold more potent inhibitory effect on T_EM_ cells relative to T_reg_ cells resulting in a non-monotonic dose-response relationship for the T_reg_:T_EM_ ratio, reaching maximum at ~ 3 mg/kg/q2w dose. Target-mediated elimination led to nonlinear PK with accelerated clearance at lower doses due to high affinity binding and rapid clearance of the drug-target complex. Doses ≥ 3 mg/kg q2w result in sustained PF-06342674 concentrations higher than the concentration of cellular IL-7 receptor and, in turn, maintain near maximal receptor occupancy over the dosing interval. The results provide important insight into the mechanism of IL-7Rα blockade and immunomodulatory activity of PF-06342674 and establish a rational framework for dose selection for subsequent clinical trials of PF-06342674. Furthermore, this analysis serves as an example of mechanistic modeling to support dose selection of a drug candidate in the early phases of development.

**Electronic supplementary material:**

The online version of this article (10.1208/s12248-019-0401-3) contains supplementary material, which is available to authorized users.

## INTRODUCTION

Type 1 diabetes (T1D) is an autoimmune disease characterized by T cell–mediated destruction of the insulin-secreting beta cells, resulting in insulin deficiency and hyperglycemia [1]. The standard-of-care treatment is daily insulin injections in an effort to normalize blood glucose levels throughout the day and ultimately to prevent long-term diabetic complications including diabetic retinopathy, nephropathy, and neuropathy. Despite the improvements in management of diabetes, there are no approved therapies which modulate the course of disease, and a large proportion of subjects with T1D fail to achieve optimal glycemic control[2].

Disease progression in T1D can be quantified as a loss of pancreatic beta cell function over a period of years, approximately 70% of which is prior to appearance of hyperglycemia and glycosuria [3]. The destruction of beta cells is a consequence of direct cytotoxicity mediated by beta cell–reactive T cells. The autoreactive T cell response in T1D has been attributed in part to a loss of peripheral tolerance caused by a relative increase in the ratio of effector memory (T_EM_) compared with regulatory T cell (T_reg_), which stem from both genetic and environmental factors [1]. The T cell subsets, along with their relative ratios, have been used as surrogate biomarkers in early phase trials in T1D. Enhanced ratios of T_reg_ to potentially pathogenic T_EM_ cells have been associated with preservation of beta cell function in subjects with new onset T1D [4,5].

The IL-7 receptor-α (IL-7Rα) gene is one of the several genetic loci that has been linked to susceptibility to T1D [6]. IL-7Rα is expressed both as a soluble receptor and a membrane bound receptor on the surface of thymocytes and T cells, both of which bind the cytokine IL-7 [7,8]. IL-7 is critical for T cell development and function, particularly the survival and activity of CD4+ and CD8+ T_EM_ cells [9,10]. Independent preclinical studies in the non-obese diabetic (NOD) mouse evaluating monoclonal antibodies (mAb) targeting IL-7Rα have demonstrated reversal of autoimmune diabetes by promoting inhibition of diabetogenic T_EM_ cells and consequently altering the balance of T_reg_ and T_EM_ cells [11,12].

Notably, a number of agents that were effective in prevention and reversal of diabetes in NOD mice have subsequently failed to show efficacy (e.g., GAD65 (alum), sitagliptin and lansoprazole, anti-IL-1, anti-thymocyte globulin (ATG)), or were only partially effective (Fc receptor nonbinding anti-CD3 mAbs and anti-CD20 mAb) in clinical trials [13]. These failures point to key clinical development challenges including a narrow window of time for treatment of subjects diagnosed with T1D, given their declining beta cell function, as well as an insufficient understanding of dose-response (DR) relationships in early clinical trials [1]. Since early clinical trials are not usually long enough nor are they powered to detect changes in clinical response endpoints such as C-peptide, it is essential to establish a model-based framework to characterize and delineate the measures of pharmacokinetics (PK), target engagement, and immunomodulatory activity obtained from the early clinical trials and to explore potential dose and exposure-response relationships to guide design of subsequent trials.

PF-06342674 is a fully human immunoglobulin G1 (IgG1) mAb that binds to IL-7Rα blocking cognate binding of IL-7 and inhibiting IL-7Rα signaling and function. PF-06342674 has previously been evaluated following single ascending doses by either subcutaneous (SC) or intravenous (IV) routes of administration in healthy volunteers (ClinicalTrials.gov, NCT01740609), and following multiple ascending doses (MAD) administered by subcutaneous injection in adults with T1D (ClinicalTrials.gov, NCT02038764) [14]. In the MAD study, the safety and tolerability of multiple SC doses of PF-06342674 were evaluated in adults diagnosed with T1D within 2 years of study entry. Additional study objectives included characterization of PK and exposure-response relationships of PF-06342674 on IL-7Rα target engagement and PD biomarkers. For this purpose, the analysis described herein was carried out utilizing two target engagement biomarkers (total soluble IL-7 receptor measured by enzyme-linked immunosorbent assay (ELISA) and cellular IL-7 receptor occupancy (RO) measured by flow cytometry) and absolute count of two surrogate markers (T_reg_ and T_EM_) measured by flow cytometry. The goals were to develop models describing the population and individual PK/PD profiles; to identify potential sources of PK nonlinearity; and to quantify PK/PD variability. The resulting model could then be used to gain quantitative understanding of the PK/PD relationships and provide simulations for doses not evaluated in the study (e.g., 6 mg/kg q2w). Altogether, the results would be used for dose selection for a proof-of-concept trial aimed at evaluation of clinical response endpoints in subjects with T1D.

## MATERIALS AND METHODS

### Study Design

The study was a phase 1b, multi-center, within cohort randomized, double-blind (sponsor-open), placebo-controlled study in adults with T1D. Additional details of the study design, as well as the safety and immunogenicity results of this study, are presented separately [14]. Briefly, eligible participants were adults (aged ≥ 18 years) with a diagnosis of T1D based on the American Diabetes Association criteria within 2 years of randomization; confirmation of at least one T1D-related autoantibody (i.e., GAD, ICA512/IA2, anti-ZnT8, or insulin autoantibodies (provided insulin therapy of less than 14-day duration)) present either at screening or documented history within 2 years of randomization; peak stimulated C-peptide levels ≥ 0.15 ng/mL measured during a mixed-meal tolerance test (MMTT) prior to randomization; body mass index (BMI) of 18.5 to 32 kg/m^2^; and total body weight ≥ 40 kg and ≤ 120 kg. The sample size was not determined based on statistical power considerations. Each cohort was targeted to enroll approximately 10 subjects with an 8:2 ratio of active drug to placebo for cohorts 1 (1 mg/kg *vs.* placebo q2w), 2 (3 mg/kg *vs.* placebo q2w), and 3 (8 mg/kg *vs.* placebo q2w). Cohort 4 (6 mg/kg *vs.* placebo q1w) was targeted to enroll approximately 5 subjects with a 4:1 ratio.

The treatment period was 10 weeks and subjects were followed up to 18 weeks for PK, PD, and safety assessments. For cohorts 1 through 3 (q2w), PF-06342674 was administered via SC injection on days 1, 15, 29, 43, 57, and 71. Serum PK samples were collected for measurement of PF-06342674 at pre-dose and 1, 4, and 48 h post dose on days 1 and 71; on days 3, 8, 15, 29, 43, 57, 73, 78, and 85 of the treatment period; and on days 92, 99, 113, and 127 during the follow-up period. Serum biomarker samples were collected for measurement of soluble IL-7Rα (sIL7Rα) at pre-dose and 1 and 48 h post dose on day 1. For cohort 4 (q1w), PF-06342674 was administered via SC injection on days 1, 8, 15, 22, 29, 36, 43, 50, 57, 64, 71, and 78. Serum PK samples were collected for measurement of PF-06342674 at pre-dose and 1, 4, and 48 h post dose on days 1 and 78; on days 3, 8, 15, 29, 43, 57, 71, 80, and 85 of the treatment period; and on days 92, 99, 113, and 127 during the follow-up period. Serum and whole blood biomarker samples were collected at a limited set of time points that were time-matched with PK sample collection times according to the dosing regimen–specific collection scheme.

### Assays

Total (free and bound) serum PF-06342674 concentrations were analyzed using a validated, sensitive, and specific sandwich ELISA assay with a lower limit of quantification (LLOQ) of 75.0 ng/mL and upper limit of quantification (ULOQ) of 1500 ng/mL. Intra-batch accuracy (%CV) and precision (%RE) were − 9.87% to 29.9% and ≤ 16.2%, respectively. Inter-batch accuracy and precision were 4.89% to 14.4% and ≤ 13.4%, respectively. Total (free and bound) sIL7Rα concentrations were measured using a validated electrochemiluminescent assay (ECLA) with a LLOQ and ULOQ of 0.7 ng/mL and 241 ng/mL, respectively. Accuracy and precision were 0% to 3.85% and ≤ 15.7%, respectively.

Lymphocyte populations were assessed by flow cytometry using fluorochrome-conjugated antibodies directed against specific cell surface markers to enumerate different subsets. The IL-7Rα RO was measured on CD3+ T cells and reported as a relative percent of baseline using an assay validated based on similar methodology to that described previously [15]. Intra-assay precision was 3.56% and intra-subject variation was 6.34%. T effector memory (CD4+CCR7-CD45RA-) and T regulatory cells (CD4+ Foxp3+) were measured as absolute counts (cells/μL). Intra-assay precision was within 5% and intra-subject variation was within 6%.

### Model Development

The objectives of model development were to characterize the PK and target engagement biomarkers to gain insight into the PK and its relationship to IL-7Rα blockade and to establish the dose-response relationship of key immunomodulatory endpoints to inform dose selection. To achieve the first objective, mechanism-based model development was performed using a simultaneous approach to fit to individual patient profiles consisting of antibody concentration, total soluble receptor, and receptor occupancy data measured over time in each patient during the treatment and follow-up periods. To achieve the second objective, dose-response model development was performed to characterize the drug effect of PF-06342674 on lymphocyte populations and their ratios that have been used as surrogate biomarkers in early phase trials in T1D. Independent and combined T_EM_ and T_reg_ DR models were evaluated to assess the impact of potential correlation between the two cell populations. The NONMEM control files for all three models are included in the supplemental files.

The typical values for intercompartmental clearance (1.1 L/h) and bioavailability (50%) were fixed in the model to the estimates previously obtained in healthy volunteers (HV) administered PF-06342674 as an intravenous (IV) infusion (unpublished results) and subcutaneous (SC) injection. This choice was based on the notion that these parameters were not identifiable in the present study in T1D subjects, of which the inclusion would potentially add uncertainty to the overall parameter estimation and the use of the typical values would not affect achieving the goal of the model development in the present study.

Assessment of model adequacy was guided by graphical and numerical approaches including a successful minimization of the objective function and plausible parameter estimates; a successful covariance step in NONMEM and reasonable precision (e.g., structural parameters less than ~ 50%) of the parameter estimates calculated as the magnitude of the relative standard errors (RSE%); and visual inspection of standard goodness-of-fit (GOF) plots.

#### Structural Model

**Fig. 1:**
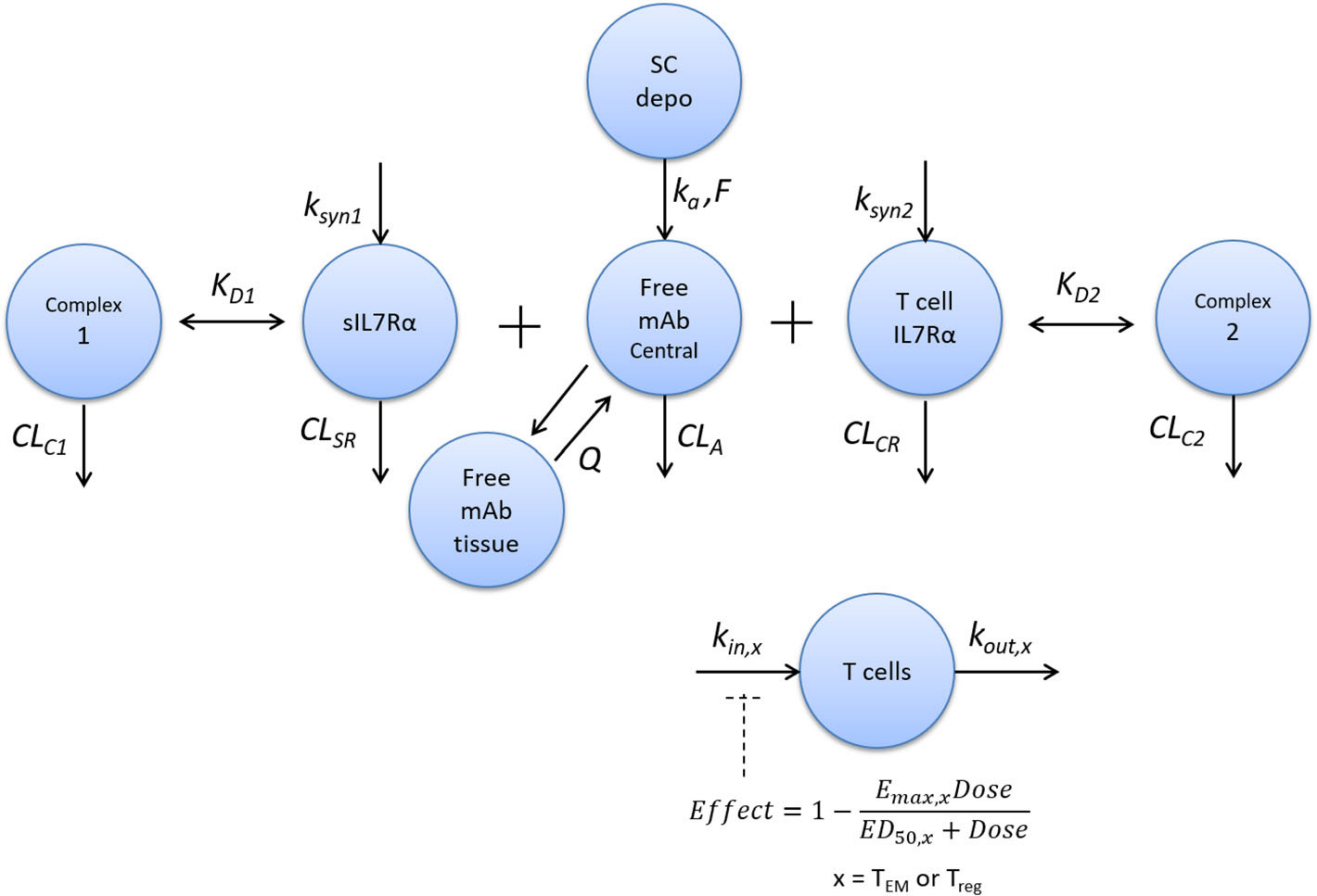
The structure of the model for the population pharmacokinetics and target engagement biomarkers of PF-06342674, a humanized mAb against IL7 receptor-α, and immunomodulatory activity in patients with type 1 diabetes. *CL*_*A*_ free antibody clearance, *CL*_*C1*_ clearance of drug-receptor complex 1, *CL*_*C2*_ clearance of drug-receptor complex 2, *CL*_*CR*_ clearance of free receptor on T cells, *CL*_*SR*_ clearance of free soluble receptor, *ED*_*50*_ antibody concentration required to achieve the half maximum effect, *E*_*max*_ maximum effect of antibody, *F* bioavailability of SC dose, *k*_*a*_ absorption rate constant, *K*_*D1*_ dissociation constant for drug-receptor complex 1, *K*_*D2*_ dissociation constant for drug-receptor complex 2, *k*_*in*_ synthesis rate of T cell subset, *k*_*out*_ degradation rate of T cell subset, *k*_*syn1*_ synthesis rate of soluble receptor, *k*_*syn2*_ synthesis rate of receptor on T cells, *Q* intercompartmental clearance.

The structure of the mechanism-based model is shown in Fig. 1. Absorption of PF-06342674 into the blood stream following SC administration was described by a first-order process, similar to previous population PK models describing various mAbs [16]. More complex characterization of the convective uptake of the antibody by the lymphatics into the circulation was not attempted due to the lack of available PK data in tissue or lymphatics necessary to describe these processes.

A two-compartment disposition model including distribution into peripheral compartment with a target-independent elimination pathway in the central compartment was retained as the base structural model as it was previously determined from data collected in HVs administered PF-06342674 following both SC and IV administration (unpublished results). In addition to a target-independent pathway of elimination, the model described the binding of PF-06342674 to either soluble or membrane-bound receptors with subsequent elimination of the resulting complexes using the quasi-equilibrium (QE) target-mediated drug disposition approximation [17,18] and assuming that all binding, internalization, and degradation take place in the central compartment. Binding or proteolytic elimination of the mAb in the lymphatics and lymph nodes, which have some potential for these activities given they are areas where T cells are concentrated, was not characterized by the model due to the lack of data to inform these processes. The QE approximation assumes that binding and dissociation of the complex are at equilibrium which is plausible because the rates are orders of magnitude faster than other processes. The binding of antibody to either soluble or cellular receptor was reversible, such that the complex may dissociate while undergoing internalization and degradation. Free antibody may also be eliminated by non-target-mediated pathways typical of protein (IgG) degradation facilitated by reticuloendothelial cells. Turnover of free soluble and cellular receptors was described as first-order synthesis of the receptor(s) and elimination either as free receptor or via the antibody-receptor complex. For convenience, the two-compartment model with the target-mediated drug disposition approximations for the drug-target engagement interactions is referred below as the TMDD model.

The equations describing the TMDD model were derived (see supplement file) similar to Hayashi *et al.* [18] and adapted to the situation of a single antibody binding to two targets, shown as follows:

1 $$ \frac{d{A}_1}{dt}={k}_a{A}_5+Q\left(\frac{A_2}{V_p}-\frac{FAB}{V_c}\right)-\frac{CL_A}{V_c} FAB-\frac{CL_{C1}}{V_c}{CPX}_1-\frac{CL_{C2}}{V_c}{CPX}_2 $$

2 $$ \frac{d{A}_2}{dt}=Q\left(\frac{FAB}{V_c}-\frac{A_2}{V_p}\right) $$

3 $$ \frac{d{A}_3}{dt}={k}_{syn1}-\frac{CL_{SR}}{V_c} FSR-\frac{CL_{C1}}{V_c}{CPX}_1 $$

4 $$ \frac{d{A}_4}{dt}={k}_{syn2}-\frac{CL_{CR}}{V_c} FCR-\frac{CL_{C2}}{V_c}{CPX}_2 $$

5 $$ \frac{d{A}_5}{dt}=-{k}_a{A}_5 $$

6 $$ {CPX}_1=\frac{1}{2}\left[\left({K}_{D1}{V}_C+{A}_1+{A}_3-{CPX}_2\right)-\sqrt{{\left({K}_{D1}{V}_C+{A}_1+{A}_3-{CPX}_2\right)}^2-4\left({A}_1-{CPX}_2\right){A}_3}\right] $$

7 $$ {CPX}_2=\frac{1}{2}\left[\left({K}_{D2}{V}_C+{A}_1+{A}_4-{CPX}_1\right)-\sqrt{{\left({K}_{D2}{V}_C+{A}_1+{A}_4-{CPX}_1\right)}^2-4\left({A}_1-{CPX}_1\right){A}_4}\right] $$

8 $$ FAB={A}_1-{CPX}_1-{CPX}_2 $$

9 $$ FSR={A}_3-{CPX}_1 $$

10 $$ FCR={A}_4-{CPX}_2 $$

Initial conditions for these equations were set to the following:

*A*_1_(0) = 0; *A*_2_(0) = 0; *A*_3_(0) = *BL*_*SR*_; *A*_4_(0) = *BL*_*CR*_; *A*_5_(0) = Dose;

Here, *A*_1_ = total antibody amount in central compartment; *A*_2_ = free antibody in peripheral tissue; *A*_3_ = total soluble receptor in central compartment; *A*_4_ = total cellular receptor in central compartment; *A*_5_ = total drug amount in the depot compartment; *BL*_*CR*_ = baseline concentration of cellular receptor; *CL*_*A*_ = clearance of the free antibody; *CL*_*C1*_, clearance of drug-receptor complex; *CL*_*C*2_, clearance of drug-receptor complex 2; *CL*_*CR*_, clearance of free receptor on T cells; *CL*_*SR*_, clearance of free soluble receptor; *CPX*_*1*_ = concentration of the antibody:soluble receptor complex; *CPX*_*2*_ = concentration of the antibody:cellular receptor complex; FAB = free antibody in central compartment; FCR = free cellular receptor in central compartment; FSR = free soluble receptor in central compartment; *K*_*D1*_, dissociation constant for drug-receptor complex 1; *K*_*D2*_, dissociation constant for drug-receptor complex 2; *k*_syn1_, synthesis rate of soluble receptor; *k*_syn2_, synthesis rate of receptor on T cells; *Q*, intercompartmental clearance; *V*_c_ = volume of the central compartment; *V*_p_ = volume of the peripheral compartment.

#### Dose-Response Model

The dose-response relationship in terms of immunomodulatory activity of PF-06342674 was described by a cellular turnover indirect response model, similar to a model which characterized the DR relationship of an S1P_(1)_ modulator on reduction of T, B, and NK cells [19]. The turnover of lymphocytes was characterized by a zero-order input rate constant (*k*_in_) and a first-order elimination rate constant (*k*_out_). The drug effect was characterized by an *E*_max_ model where increasing the dose of PF-06342674 would result in a reduction in the input rate. For DR indirect effect models, the explicit solution for differential equations describing inhibition of stimulation models has been described [20] Solving for response *R*(*t*), where *R* = T_EM_ or T_reg_, the algebraic expression is

11 $$ R(t)={R}_0\left\{{e}^{-{k}_{\mathrm{out}}\cdotp t}+\left(1-\frac{E_{\mathrm{max}}\mathrm{Dose}}{ED_{50}+\mathrm{Dose}}\right)\left[1-{e}^{-{k}_{\mathrm{out}}\cdotp t}\right]\right\} $$

where

12 $$ {R}_0/{k}_{\mathrm{out}}={k}_{\mathrm{in}} $$

and at steady state, Eq. 11 can be further reduced as follows:

13 $$ R\left(t=\infty \right)={R}_0\left(1-\frac{E_{\mathrm{max}}\mathrm{Dose}}{ED_{50}+\mathrm{Dose}}\right) $$

Here, *BL*_EM_ = baseline concentration of T_EM_ lymphocytes; *BL*_Treg_ = baseline concentration of T_reg_ lymphocytes; *ED*_50_ = dose required to achieve the half maximum effect; *E*_max_ = maximum drug effect; *k*_in_ = synthesis rate of T cell subset; *k*_out_ = degradation rate of T cell subset; *R*_0_ = *BL*_EM_ for the T_EM_ model, *BL*_TR_ for the T_reg_ model.

#### Statistical Model

Combinations of interindividual variability (IIV) in various PK/PD parameters were considered and evaluated in an exploratory step of model building. In all cases, IIV on individual parameters was described by the log-normal distribution

$$ {P}_i=\hat{P}\exp \left({\eta}_i\right) $$

where *P*_*i*_ is the estimated parameter value for the individual *i*, $\hat{P}$ is the typical population value of the parameter, and *η*_*i*_ denotes the inter-individual random effect accounting for the *i*th individual’s deviation from the $\hat{P}$ having zero mean and variance *ω*^*2*^ on the natural logarithm scale.

The multivariate vector of inter-individual random effects (across parameters within each individual) has variance-covariance matrix *Ω*. A full block covariance matrix for the inter-individual random effects (*Ω*) was estimated for PK parameters.

Residual variability was described using an additive, proportional, or combined additive and proportional error model as described below.

$$ {C}_{ij}={\hat{C}}_{ij}\left(1+{\upvarepsilon}_{p, ij}\right)+{\varepsilon}_{a, ij} $$

where *C*_*ij*_ is the *j*th measured observation in individual *i*, ${\hat{C}}_{ij}$ is the corresponding model-predicted value, and *ε*_*a,ij*_ and *ε*_*p,ij*_ the corresponding additive and proportional error, respectively, normally distributed with mean 0 and variance *σ*^*2*^.

#### Model Evaluation

Standard goodness-of-fit diagnostic plots were examined to aid evaluation of model adequacy, comparing the observations with individual and population model predictions, as well as residual plots to assess adequacy of the random effects model. Prediction-corrected visual predictive checks (pcVPCs) comparing the empirical with the model-predicted 10th, 50th, and 90th percentiles were used to assess the predictive performance of the final models [21].

#### Simulations

The final models were combined into a single simulation model and simulations were performed to illustrate PK/PD time courses for the doses used in this study, as well as intermediate dose levels (see supplemental file for *mrgsolve* model and simulation code). Assessment of the DR for T_EM_, T_reg_, and T_reg_:T_EM_ ratio endpoints, including uncertainty in parameter estimates, was conducted using 1000 parameter sets obtained from a nonparametric bootstrap and resampling with replacement using the final DR models.

#### Software

Population PK/PD and DR analysis was conducted via nonlinear mixed-effects modeling with NONMEM software, version 7.4.3 (ICON Development Solutions, Ellicott City, Maryland). Visual predictive checks and bootstrapping were performed using Perl-speaks-NONMEM (PsN) version 4.2.0 [22]. Data sets formatting and post-processing of model fitting and simulation outputs were performed using R version 3.2.2 (R Foundation, Vienna, Austria). Simulations were conducted in R using the *mrgsolve* package [23].

## RESULTS

### Subjects and Data Set Characteristics

A total of 37 subjects enrolled in the study; 36 subjects were included in the analysis of T lymphocyte (T_EM_, T_reg_); and 26 subjects were included in the analysis of PK, sIL7Rα, and Free RO. One subject discontinued after the first dose was excluded from both analyses. Three subjects were excluded from the analysis of PK, sIL7Rα, and Free RO due to issues related to PK or missing Free RO measurement at baseline. Subjects treated with placebo (*n* = 7) were excluded from the analysis of PK, sIL7Rα, and Free RO. A breakdown of the number of subjects by cohort and the number of PK and PD measurements is provided in Table I. The overall demographics of subjects enrolled in the study are described elsewhere [14].

**Table I:** Summary of Observations, Number of Subjects and Baseline Concentrations by Dose Group

	Placebo	1 mg/kg q2w	3 mg/kg q2w	8 mg/kg q2w	6 mg/kg q1w	Total
TMDD model population						
Number of subjects	0	5	8	8	5	26
		No. of obs				
PF-06342674 concentrations	–	91	150	149	84	474
IL-7Rα RO on T cells	–	62	101	99	59	348
		No. of obs, mean (CV)				
Soluble IL-7Rα receptor, ng/mL	–	66, 12.3 (38)	109, 13.6 (25)	109, 14.6 (50)	64, 15.5 (48)	321, 14 (39)
T lymphocyte model population						
Number of subjects	7	8	8	8	5	36
	No. of Obs, Mean (CV)					
Effector memory, cells/μL	81, 78.8 (47)	89, 61.6 (39)	86, 46.7 (47)	88, 98.3 (58)	55, 104 (27)	399, 76.4 (52)
T regulatory, cells/μL	82, 44.3 (41)	88, 45.7 (48)	87, 46.5 (31)	90, 63.3 (50)	58, 50.6 (30)	405, 50.3 (41)

*No.* number, *Obs* observations, *CV* coefficient of variation (%), *RO* receptor occupancy

The baseline concentrations of sIL7Rα and absolute counts of T_EM_ and T_reg_ are shown in Table I. Overall, the level of sIL7Rα was similar across treatment groups, ranging from 12 to 16 ng/mL, and T_reg_ counts, ranging from 44 to 63 cells/μL. Absolute counts of T_EM_ were higher in 8 mg/kg q2w and 6 mg/kg q1w treatment groups, ranging 98 to 104 cells/μL, compared with the placebo, 1 mg/kg q2w and 3 mg/kg q2w groups, ranging from 47 to 79 cells/μL. To retain information on between-group variability of the cell populations, absolute counts of T_EM_ and T_reg_ populations were used in the modeling rather than normalizing change from baseline.

### TMDD Model

In general, PF-06342674 exhibits nonlinear PK, with faster elimination observed at lower concentrations suggestive of target-mediated drug disposition. Total sIL7Rα increased post-treatment in a dose-dependent manner and returned to baseline consistent with PK time course. Likewise, nearly complete saturation of the receptor was achieved at the 3 mg/kg q2w level or higher. Effector memory and T_reg_ cell subsets were reduced in a gradual fashion over approximately 4 to 8 weeks and did not completely return to baseline during the follow-up period.

**Fig. 2:**
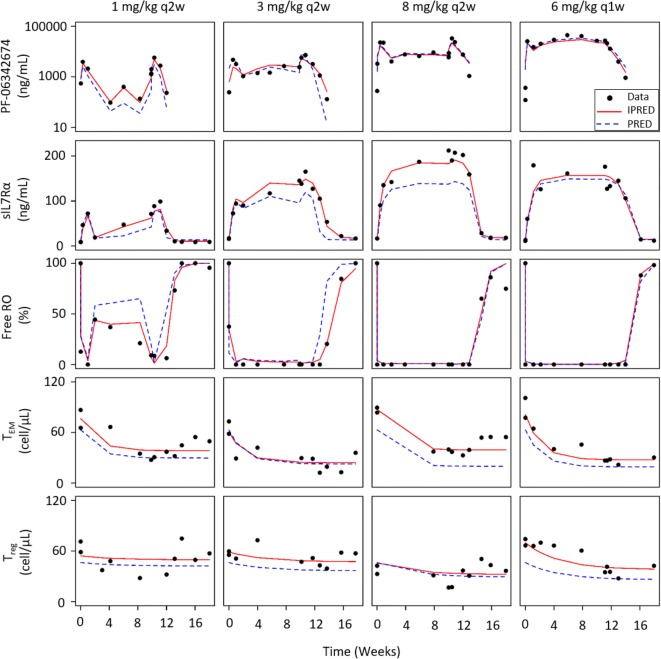
Example of individual model predictions (IPRED), population predictions (PRED), and observations (data) for the five endpoints in the model. A single representative subject from each of the four dose cohorts is shown. Individual predictions for all subjects are shown in Figures S3-S7

**Fig. 3:**
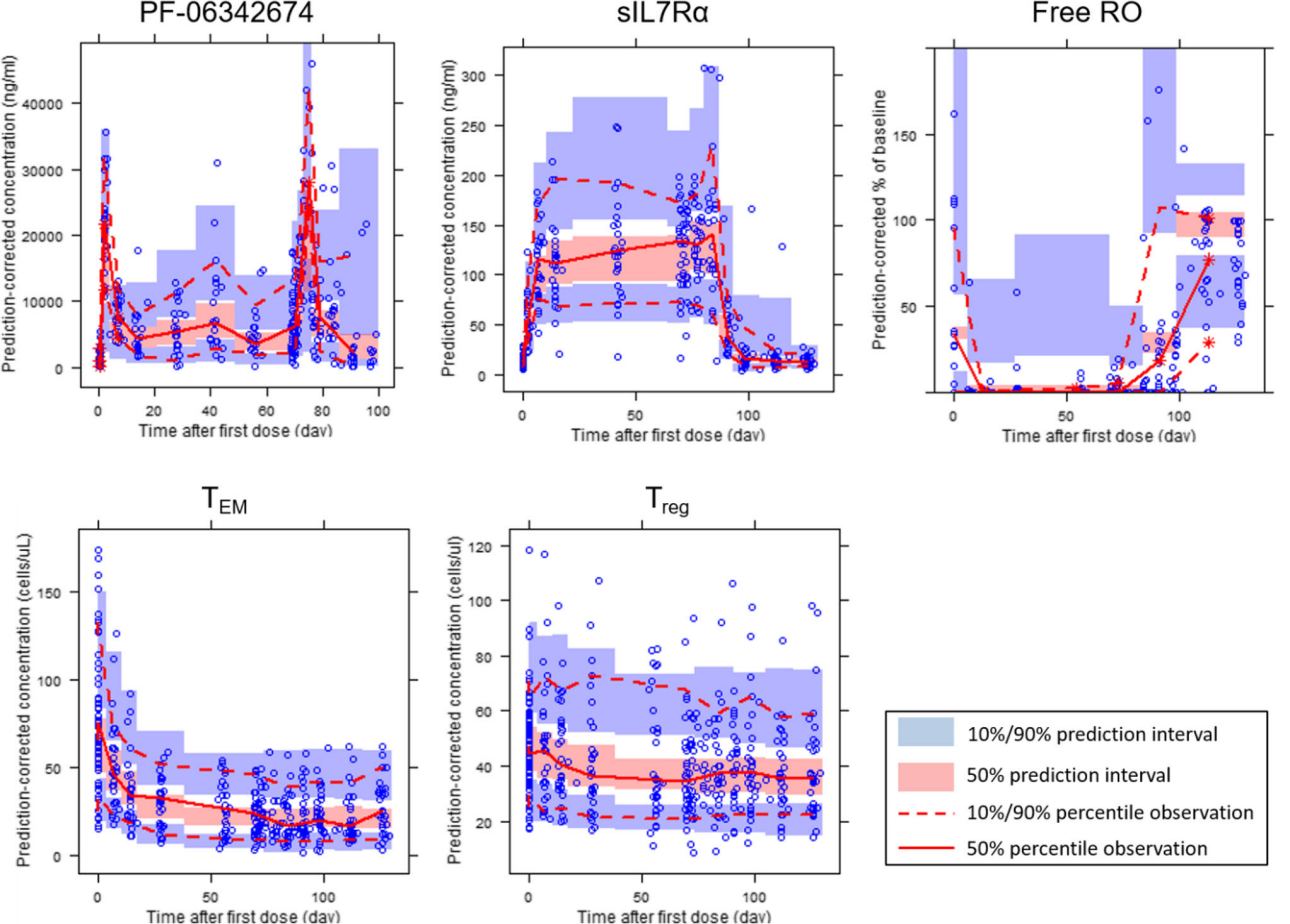
Prediction-corrected visual predictive check (pcVPC) comparing the empirical 10th, 50th, and 90th percentiles with the simulated 10%, 50%, and 90% prediction intervals (PI) for serum concentrations of PF-06342674 and sIL7Rα, percentage Free RO, and absolute counts of T_EM_ and T_reg_

Correlation between PK and target engagement biomarkers was explicitly stated in the TMDD model equations (see “Materials and Methods”) which captured the relationship between the concentration-time profiles at the individual-subject level (Fig. 2). The turnover models provided a good representation of T cell dynamics and the inhibitory activity of PF-06342674 on this process. Overall, the models fit the data well capturing both the central tendency and distribution for PK, sIL7Rα, Free RO, T_EM_, and T_reg_ measures (Fig. 3). Goodness-of-fit plots indicated good agreement between population- or individual-predicted concentration and observed concentration as well as the random distribution of conditional weighted residuals (Figures S8-S12). While pcVPCs for Free RO indicated slight overprediction, inspection of VPCs stratified by dose (Figure S2) and individual predictions and observations (Fig. 2, Figure S5) indicated the model adequately captured the individual Free RO *versus* time profiles.

The parameters estimated from the mechanism-based model are summarized in Table II. Most parameters were estimated with good precision (RSE ≤ 30%). Following SC administration, PF-06342674 was slowly absorbed via first-order kinetics at a rate of 0.21 day^−1^ consistent with the median (0.21 day^−1^) determined from analysis of various mAbs characterized using population PK methods [16].

**Table II:** Population Parameter Estimates for the TMDD Model

Parameter	Units	Description	Estimate (RSE%)	IIV
*CL*_A_	L/day	Clearance of antibody	0.999 (9)	42.5 (0.08)
*V*_C_	L	Central volume of distribution	1.10 (7)	7.3 (22)
*V*_P_	L	Peripheral volume of distribution	5.28 (22)	–
*Q*	L/day	Inter-compartmental clearance	1.1^*a*^	–
*F*	%	Subcutaneous bioavailability	50^*a*^	–
*k*_A_	Day^−1^	Subcutaneous absorption rate	0.211 (8)	31.3 (7)
*CL*_SR_	L/day	Clearance of sIL7Rα	2.24 (23)	–
*V*_R_	L	Volume of sIL7Rα	= *V*_C_	–
*CL*_C1_	L/day	Clearance of the mAb:sIL7Rα complex	0.196 (21)	–
*K*_D1_	nM	Dissociation constant of mAb:sIL7Rα	0.779 (59)	–
*BL*_SR_	nM	Baseline concentration of sIL7Rα	0.45 (14)	35.1 (0)
*CL*_CR_	L/day	Clearance of the cIL7Rα	10.4 (26)	–
*CL*_C2_	L/day	Clearance of the mAb:cIL7Rα complex	= *CL*_CR_	–
*K*_D2_	nM	Dissociation constant of mAb:cIL7Rα	0.450 (20)	–
*BL*_CR_	nM	Baseline concentration of cIL7Rα	1.37 (13)	–
Residual error (ε)				
*σ*_1_	%	Proportional error, PK	0.434 (6)	–
*σ*_2_	%	Additive error, RO	18.2 (30)	–
*σ*_3_	%	Proportional error, sIL7Rα	0.150 (8)	–

*RSE* relative standard error, *IIV* interindividual variability expressed as % coefficient of variation (% *η*-shrinkage), *sIL7Rα* soluble IL7 receptor α, *cIL7Rα* IL7 receptor α on T cells, *mAb* monoclonal antibody. Dashes indicate data not computed

^*a*^Fixed to the typical value estimated from healthy volunteers administered SC or IV PF-06342674

PF-06342674 distributed into central and peripheral compartments, consistent with other mAbs that exhibit bi-phasic distribution, particularly with data obtained from subjects receiving intravenous injections [16]. Bioavailability and distribution parameter estimates, obtained from healthy volunteers, were used to simplify the model building process and to address identifiability issues in the absence of IV information in this subject population. It was assumed these parameter estimates are similar across the two populations, a reasonable assumption given the subject population was generally in good health. The estimate of steady-state volume of distribution (*V*_ss_ = *V*_c_ + *V*_p_ = 6.4 L) was consistent with the estimates of distribution volume for endogenous IgG (6.2 L) (16,24). However, the estimate of distribution volume in the central compartment (*V*_c_) was lower (1.1 L) relative to the range determined for various mAbs (2.4 to 5.5 L) [16]. The fixed value of inter-compartmental clearance (1.1 L/day), estimated from healthy subjects (unpublished results), was consistent with the median value (0.79 L/day) from recent analysis [16].

**Fig. 4:**
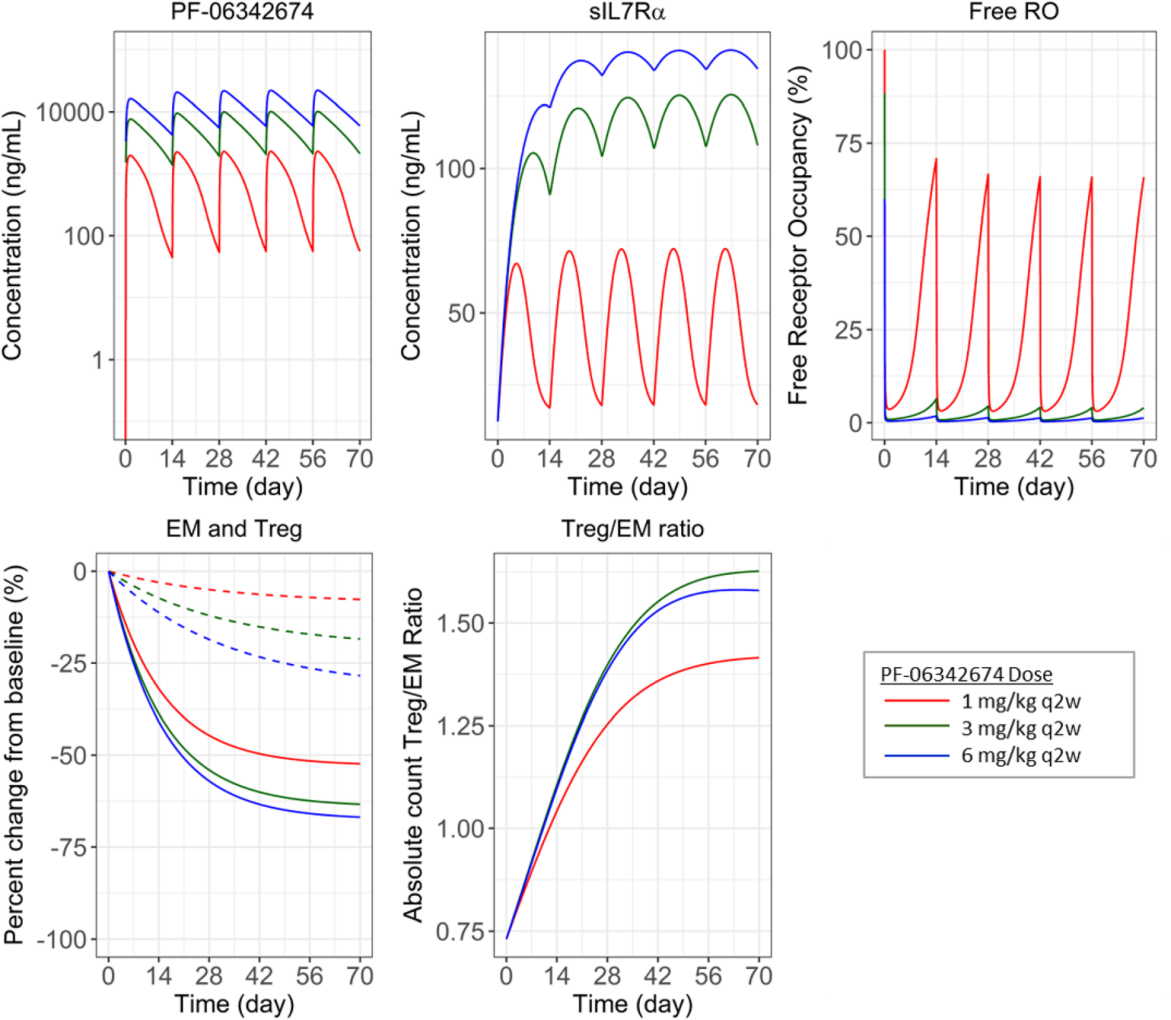
Simulations following q2w SC doses of 1 mg/kg, 3 mg/kg, and 6 mg/kg. Shown are the profiles of serum concentrations of PF-06342674 and sIL7Rα, percentage Free RO from the TMDD model, and absolute counts (cells/μL) of T_EM_ and T_reg_ and T_reg_:T_EM_ ratio from the dose-response model

Evidence of target-mediated drug disposition was observed by high-affinity binding of PF-06342674 to the cellular receptor (*K*_D2_ = 0.450 nM) and a 10-fold more rapid clearance of the resulting complex (*CL*_C2_ = 10.4 L/day) compared with the free mAb clearance (*CL*_A_ = 1 L/day). The estimated baseline concentration of cellular receptor (BL_CR_) was 1.37 nM, and at a dose of 1 mg/kg q2w, the PK profile of free mAb and mAb:cIL7Ra complex confirms the predominant elimination pathway utilized at this dose level is target-mediated, with free mAb falling below the BL_CR_ concentration by post-treatment day 8, and from days 8 to 14 post-treatment, the predominant species is the complex (Supplemental Figure 1). At a higher dose of 3 mg/kg q2w, the concentration of free mAb ≈ total mAb over the 14-day dosing interval, indicating the predominant elimination pathway is target-independent, and the resulting total mAb (PF-06342674) PK profiles are mostly linear. The RO profiles reflect these findings such that following treatment with 1 mg/kg q2w, near-maximal RO is rapidly achieved (Free RO < 2%), but by day 8 Free RO ~ 8% and by day 14 post-treatment Free RO ~ 68% (34% RO). At 3 mg/kg q2w, near-maximal RO (98%) is maintained over the entire dosing interval (Fig. 4).

Accumulation of the total soluble receptor, shown as increasing concentrations following treatment with PF-06342674, resulted as a consequence of slower clearance of the PF-06342674:sIL7Rα complex (CL_C1_) compared with the free soluble receptor clearance (CL_SR_). Inclusion of IIV on *CL*_A_, *V*_C_, *k*_A_, and *BL*_SR_ resulted in a parsimonious model that was able to capture the variability between subjects and provided an adequate fit to individual profiles (Fig. 2). The IIV was moderate for *CL*_A_ (43%), absorption rate (*k*_a_, 31%), and baseline levels of soluble receptor (*BL*_SR_, 35%) and low for central volume of distribution (*V*_*C*_, 7.3%).

### Dose-Response Model

**Fig. 5:**
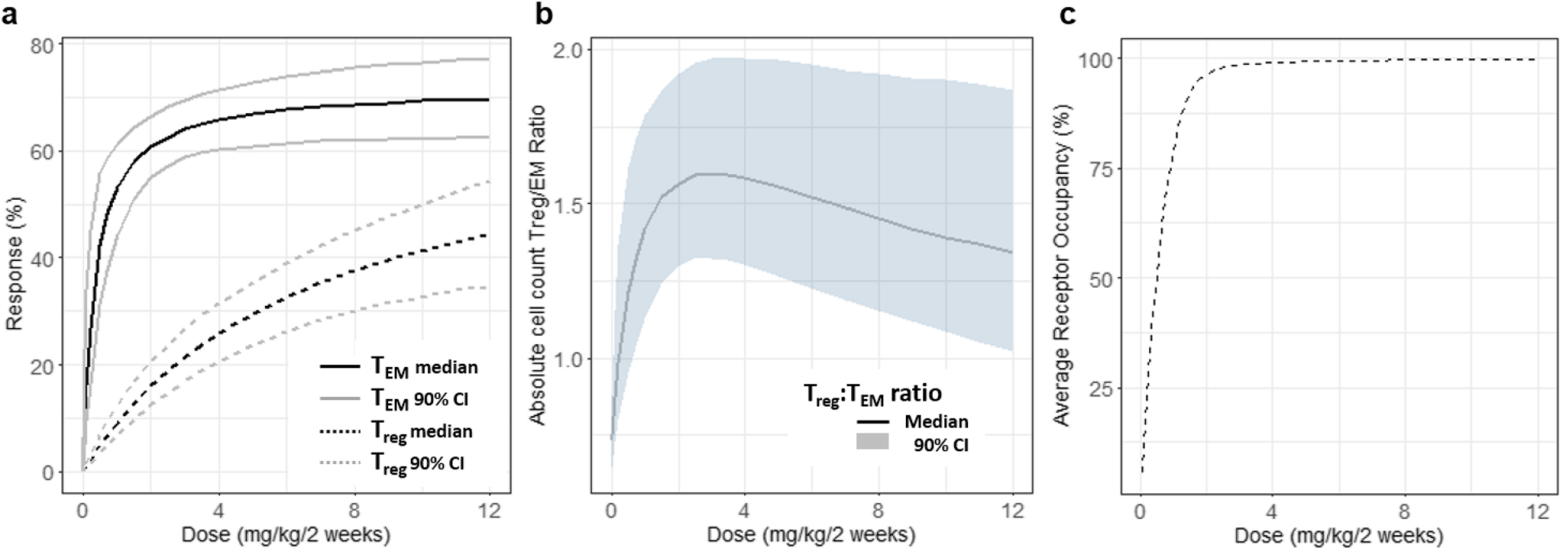
**a**–**c** Dose-response relationships, including **a**, **b** parameter uncertainty, for T_EM_, T_reg_ T_reg_:T_EM_ ratio and average receptor occupancy (%)

Both T lymphocyte populations were adequately described by the proposed DR model. A similar maximal effect (*E*_max_) was estimated for T_EM_ (72%) and T_reg_ (70%). However, as noted from the DR relationships (Fig. 5) the effect of PF-06342674 on T_EM_ cells rises rapidly from 1 to 3 mg/kg q2w; then plateaus while the effect of PF-06342674 on T_reg_ cells increases gradually over the dose range evaluated in the study. The T_EM_ population was approximately 20-fold more sensitive than T_reg_, as indicated by a lower *ED*_50_ value of 0.35 mg/kg/q2w *versus* 7.1 mg/kg/q2w, respectively, and explains the difference in the DR. Integration of T_EM_ and T_reg_ model predictions indicated the DR curve for the ratio of T_reg_:T_EM_ cell populations was non-monotonic, with an maximum ratio coinciding with the dose level which achieved near-maximal RO predicted at ~ 3 mg/kg q2w, whereas at higher doses, the ratio declines (Fig. 5).

The estimated baseline concentrations of T_EM_ and T_reg_ were 63.5 cells/μL and 45.7 cells/μL (Table III), consistent with the observed baseline concentration of 76.4 cells/μL and 50.3 cells/μL, respectively (Table I). The corresponding group mean T_reg_:T_EM_ ratio at baseline was calculated to be 0.66 (observed) and 0.73 (predicted). The disappearance rate was faster for T_EM_ (0.07 day^−1^, corresponding to a *t*_1/2_ of ~ 10 days) than for T_reg_ (0.03 day^−1^, corresponding to *t*_1/2_ of ~ 23 days). A faster input rate (calculated as *k*_in_ = *R*_0_/*k*_out_) was estimated for T_EM_ (4.2 cells/μL day^−1^) compared with T_reg_ (1.4 cells/μL day^−1^).

**Table III:** Population Parameter Estimates for the Dose-Response Models

			T_EM_		T_reg_	
Parameter	Units	Description	Estimate (RSE%)	IIV	Estimate (RSE%)	IIV
*R*_0_	Cells μL^−1^	Baseline concentration	63.1 (8.2)	41 (5)	46.2 (5.7)	33 (19)
*k*_out_	Day^−1^	First-order disappearance rate of lymphocytes	0.0665 (14)	–	0.0308 (26)	–
*E*_max_	Unitless	Maximum possible effect	0.715 (9.3)	21 (24)	0.700 (13)	27 (50)
*ED*_50_	mg/kg/2wk	Dose at half-maximum effect	0.353 (64)	–	7.06 (31)	–
*σ*_5_	%	Proportional residual error	11 (14)	–	6.1 (13)	–

*RSE* relative standard error, *IIV* interindividual variability expressed as % coefficient of variation (% *η-*shrinkage), T_EM_ effector memory T cells, *Treg* regulatory T cells

## DISCUSSION

A mechanism-based model was proposed which integrates the PK and target engagement biomarker profiles into a single mathematical framework, described by a set of algebraic and ordinary differential equations. The estimated rate of absorption and peripheral volume of distribution were consistent with previous estimates for therapeutic mAbs [16]. The estimate of the central volume of distribution (1.1 L) was lower relative to published values for mAbs (2.4 to 5.5 L) which may have been due to the lack of PK data in T1D subjects following IV administration as well as rapid binding of PF-06342674 to IL-7Rα in the central compartment. Clearance of free mAb (CL_A_ = 1 L/day) was more rapid than the value reported for mAbs which ranged from 0.2 to 0.5 L/day [16]. Estimation of the dissociation constant for drug-target binding (*K*_D_), which relied on rich PK/PD sampling schemes and measurements of drug concentration, total soluble receptor, and cellular receptor using independent bioanalytical approaches, indicated that PF-06342674 binds with high affinity to cellular (*K*_D2_ = 0.450 nM) and soluble IL-7 receptor targets (*K*_D1_ = 0.779 nM).

Clearance of the mAb:sIL7Rα complex (CL_C1_ = 0.2 L/day) was slower than free sIL7Rα (*CL*_SR_ = 2.5 L/day), which is reflected in the observation that total sIL7Rα increased considerably following each dose of PF-06342674. The observation that *CL*_C1_ is smaller than free mAb clearance is one indication that the soluble receptor pathway is not the main driver of nonlinear PK as it does not contribute in a profound way to accelerated clearance at low PF-06342674 concentrations where the drug is largely saturated by the targets. This is plausible since soluble targets often act as carriers of ligands as opposed to cellular targets which can undergo receptor-mediated endocytosis and degradation. In contrast, the clearance of the mAb:cIL7Rα complex was 10-fold more rapid than free mAb clearance suggesting elimination via the cellular receptor is likely the key source of nonlinear PK.

Model-based estimation of individual baseline concentration of cellular receptor was necessary as it was not directly measured, due to the units being post-treatment median fluorescence intensity relative to baseline. Likewise, estimation of clearance of free cIL7Rα was not supported by the data and therefore, it was assumed that mAb:cIL7Rα complex clearance was equal to free cIL7Rα clearance (i.e., *CL*_C2_ = *CL*_CR_). In contrast, measurement of the concentration of sIL7Rα at baseline provided sufficient information for estimation of both population mean and individual concentration (*BL*_SR_ = 0.45 nM, IIV_BLSR_ = 35%).

Other than BL_SR_, the sources of inter-individual variability were attributed to variation in absorption rate (31%), central volume (7%), and clearance of the free antibody (43%). Thus, the variability in RO is due predominantly to variability in PK parameters, along with residual error. Inspection of individual profiles (Fig. 2) supports this interpretation, where the direction and magnitude of the difference between population prediction and individual predictions are similar for PK and free RO. This finding suggests that in prospective studies, PK concentration could be considered a surrogate for RO, which can help reduce or eliminate the need for additional blood collections and procedure burden since RO assays often require analysis of fresh samples within 2 days of collection.

It may be anticipated that variability in absorption rate and clearance of the free antibody could be related to host factors including, for example, site of injection, body composition, and age. A systemic covariate analysis to further assess potential sources of variability in PK parameters was not conducted. Upon further development of PF-06342674, these types of additional analyses are warranted and could be used to support justification for changing from body weight–based dosing to flat-dosing which can provide greater convenience for subcutaneous administration.

A DR model was utilized to establish the DR relationship for key immunomodulatory endpoints. In mammals, generation and differentiation of T cells occur in primary lymphoid organs. All mature lymphocytes circulate through secondary lymphoid organs. T lymphocytes then transmigrate into tissue, which can be tissue-specific, and organs via a multi-step pathway [25]. The model assumes turnover of both T_reg_ and T_EM_ explained by a single rate describing the input and elimination; reduction in the absolute counts due to the inhibition on the input rate; and the effect of PF-06342674 on T cells follows an *E*_max_ relationship. Under these assumptions, the downstream modulation of lymphocyte subsets exhibited a delayed effect relative to PK and RO time courses (Fig. 2), due likely to a slower turnover rate for T_EM_ and T_reg_ relative to the half-life of PF-06342674 (*t*_1/2_~3 days). To describe this hysteresis, an indirect response model was used to characterize the time-course of immunomodulation similar to the model developed to characterize the effect of an S1P_(1)_ modulator on reduction of T, B, and NK cells [19]. The DR models adequately fit to the T cell data from subjects and retained the general mechanism of action of PF-06342674 with only four structural parameters. The effect of PF-06342674 was modeled as an inhibitory *E*_max_ function on the zero-order input rate, which reflects PF-06342674 binding to IL-7Rα and preventing T cell activation and proliferation by down-modulation of the IL-7 signaling pathway.

Summarized across the groups, absolute cell counts at baseline were higher for T_EM_*versus* T_reg_, corresponding to an overall T_reg_:T_EM_ ratio of 0.7. This value was accurately estimated by the model and was explained by the turnover rates of these cell populations (i.e., baseline = *k*_in_/*k*_out_). Comparison among the groups indicated differences in mean cell counts. Because of this, it was important to model this data in absolute cell count to retain the information between groups as this approach would yield a more accurate characterization of the underlying DR relationships. Furthermore, modeling the absolute count facilitated estimation of the T_reg_:T_EM_ ratio and in turn provided the opportunity to characterize the DR for this measure of immune activity. Following multiple SC injections of PF-06342674, a dose-dependent relationship was observed in the reversal of the T_reg_:T_EM_ ratio with a maximum observed at ~ 3 mg/kg q2w. This reversal was due to the 20-fold higher potency of PF-06342674 on T_EM_ relative to T_reg_. This was anticipated, as it has been shown that human T_reg_ expresses lower levels of IL-7Rα [26,27]. The current model suggests that doses up to those which approach maximal RO are needed for maximizing the T_reg_:T_EM_ ratio, but at higher doses approaching the *ED*_50_ for the effect of PF-06342674 on T_reg_ (7 mg/kg/q2w), the ratio starts to decline. Overall, the observed increase in the T_reg_:T_EM_ ratio provides evidence that IL-7Rα blockade may shift the balance from autoimmunity towards immune tolerance.

Lastly, we hope that this communication can serve as an example of how one can gain quantitative understanding of the PK/PD relationships for a drug candidate in early development, where the study sample size and treatment duration are limited, using insightful mechanistic modeling approaches to inform the designs of subsequent clinical trials and particularly dose selection. Such an effort may benefit from the model-based integration that takes advantage of full profiles of PK and multiple PD measures in overcoming limitations, such as small sample size, often encountered in early development. In the present study, the model-based integration of PK, target engagement biomarker, and immunomodulatory activity data offered quantitative understanding of the PK/PD relationships consistent with the postulated mechanism of IL-7Rα blockade and immunomodulatory activity of PF-06342674. This understanding strengthened not only the early evidence of therapeutic effects of the drug candidate in patients but also the confidence of using a simplified DR relationship for dose determinations, as well as utilizing simplified clinical pharmacology study procedures (relying only on PK measures) in future clinical trials. It should be also noted that the model-based integration as such can remain challenging in terms of uncertainty of the model parameter estimation, even with the use of multiple related PD measures and rich PK/PD sampling schemes. To this end, in the present study, the number of model parameters to be estimated was reduced, with fixing the intercompartmental clearance and bioavailability based on the respective prior information under the assumption that for this mAb drug candidate, these two parameters are not study-specific. Borrowing the intercompartmental clearance and bioavailability estimates from the preceding single ascending dose study in healthy volunteers using SC and IV routes of administration helped improve certainty of the model parameter estimates in the present study (data not shown), especially for those related to the target engagement and binding-mediated eliminations.

## CONCLUSION

The proposed modeling framework adequately characterized the PK, target engagement biomarkers, and immunomodulatory activity of PF-06342674, a humanized mAb against IL-7Rα in subjects with T1D. PF-06342674 binds with high affinity to cellular (*K*_D2_ = 0.450 nM) and soluble IL-7 receptor targets (*K*_D1_ = 0.779 nM), with elimination of PF-06342674 via the cellular IL-7 receptor-mediated pathway the most likely source of nonlinear PK. Inter-individual variability in PK and RO was mainly attributed to variation in the absorption rate, central volume, and clearance of the free antibody. The DR relationship characterizing the effects of PF-06342674 on the T_reg_:T_EM_ T cell ratio provides evidence that IL-7Rα blockade may shift the balance from autoimmunity towards immune tolerance. The T_reg_:T_EM_ T cell ratio increased with higher doses up to approximately 3 mg/kg q2w, after which further increasing the dose resulted in a decline in the T_reg_:T_EM_ T cell ratio due to an increasing inhibitory effect of PF-06342674 on T_reg_ numbers. Notably, the maximal effective dose with respect to T_reg_:T_EM_ T cell ratio coincides with the dose level that results in near maximal IL-7 RO. The results provide important insight into the mechanism of IL-7Rα blockade and immunomodulatory activity of PF-06342674 and establish a rational framework for dose selection for subsequent clinical trials of PF-06342674. Furthermore, this analysis serves as an example of integrating PK and multiple biomarkers using insightful mechanistic modeling approaches to gain quantitative understanding of the PK/PD relationships and support dose selection of a drug candidate in the early phases of development.

## Electronic supplementary material

ESM 1 (DOCX 732 kb)
