# Supplementary material for: Model-Based Characterization of the Pharmacokinetics, Target Engagement Biomarkers, and Immunomodulatory Activity of PF-06342674, a Humanized mAb Against IL-7 Receptor-α, in Adults with Type 1 Diabetes
Source: AAPS J. 2020 Jan 3;22(2):23. doi: 10.1208/s12248-019-0401-3 (PMC6942017; doi:10.1208/s12248-019-0401-3)
Supplement: Supplementary file 1 — (DOCX 732 kb) [file 12248_2019_401_MOESM1_ESM.docx]

**Supplemental Material**

**Derivation of TMDD model equations**

For the reaction FAB + FSR ↔ CPX_1_ at steady state:

$K_{D1}= \frac{\left[ FAB \right]\left[ FSR \right]}{\left[ {CPX}_{1} \right]}$ (S1)

For the reaction FAB + FCR ↔ CPX_2_ at steady state:

$K_{D2}= \frac{\left[ FAB \right]\left[ FCR \right]}{\left[ {CPX}_{2} \right]}$ (S2)

Free antibody, free soluble receptor and free receptor amount are described by:

$FAB= TAB-{CPX}_{1}-{CPX}_{2}$ (S3)

$FSR= TSR-{CPX}_{1}$ (S4)

$FCR= TCR-{CPX}_{2}$ (S5)

Where TAB=A(1), TSR=A(3) and TCR=A(4) in the model. Assuming all species share a similar volume (Vc), Eq. S1 and S2, which refer to concentrations, can be described by:

$K_{D1}= \frac{\frac{TAB-{CPX}_{1}-{CPX}_{2}}{V_{C}}\cdot\frac{TSR-{CPX}_{1}}{V_{C}}}{\frac{{CPX}_{1}}{V_{C}}}$ (S6)

and

$K_{D2}= \frac{\frac{TAB-{CPX}_{1}-{CPX}_{2}}{V_{C}}\cdot\frac{TCR-{CPX}_{2}}{V_{C}}}{\frac{{CPX}_{2}}{V_{C}}}$ (S7)

Solving for zero, Eq. S6 is expressed as

$0= \left( TAB-{CPX}_{1}-{CPX}_{2} \right)\cdot\left( TSR-{CPX}_{1} \right)-K_{D1}\cdot{CPX}_{1}\cdot V_{C}$ (S8)

And Eq. S7 is expressed as

$0= \left( TAB-{CPX}_{1}-{CPX}_{2} \right)\cdot\left( TCR-{CPX}_{2} \right)-K_{D2}\cdot{CPX}_{2}\cdot V_{C}$ (S9)

Multiplying through these result in:

$0= TAB\cdot TSR-{TAB\cdot CPX}_{1}-TSR\cdot{CPX}_{1}+{CPX}_{1}^{2}-TSR\cdot{CPX}_{2}+{CPX}_{1}\cdot{CPX}_{2}-K_{D1}\cdot{CPX}_{1}\cdot V_{C}$ (S10)

and

$0= TAB\cdot TCR-{TAB\cdot CPX}_{2}-TCR\cdot{CPX}_{1}+{CPX}_{1}\cdot{CPX}_{2}-TCR\cdot{CPX}_{2}+{CPX}_{2}^{2}-K_{D2}\cdot{CPX}_{2}\cdot V_{C}$ (S11)

Which can be further simplified to:

$0= TSR\cdot\left( TAB-{CPX}_{2} \right)-{CPX}_{1}\cdot\left( TAB-{CPX}_{2}+TSR+K_{D1}\cdot V_{C} \right)+{CPX}_{1}^{2}$ (S12)

and

$0= TCR\cdot\left( TAB-{CPX}_{1} \right)-{CPX}_{2}\cdot\left( TAB-{CPX}_{1}+TCR+K_{D2}\cdot V_{C} \right)+{CPX}_{2}^{2}$ (S13)

Which is of the same form as the quadratic equation:

$$0=c+bx+{ax}^{2}$$

with roots of

$$x= \frac{-b\pm\sqrt{b^{2}-4ac}}{2a}$$

For Eq. S12,

$a=1$, $b=-\left( TAB-{CPX}_{2}+TSR+K_{D1}\cdot V_{C} \right)$, $c= TSR\cdot\left( TAB-{CPX}_{2} \right)$ and $x= {CPX}_{1}$

and therefore, Eq. S12 and S13 can be expressed as

${CPX}_{1}= \frac{1}{2}\cdot\left( TAB-{CPX}_{2}+TSR+K_{D1}\cdot V_{C} \right)\pm\sqrt{\left( TAB-{CPX}_{2}+TSR+K_{D1}\cdot V_{C} \right)^{2}-4\cdot TSR\cdot\left( TAB-{CPX}_{2} \right)}$ (S14)

and

${CPX}_{2}= \frac{1}{2}\cdot\left( TAB-{CPX}_{1}+TCR+K_{D2}\cdot V_{C} \right)\pm\sqrt{\left( TAB-{CPX}_{1}+TCR+K_{D2}\cdot V_{C} \right)^{2}-4\cdot TCR\cdot\left( TAB-{CPX}_{1} \right)}$ (S15)

For Eq. S14, since CPX_1_ cannot be greater than TAB, there is one solution:

${CPX}_{1}= \frac{1}{2}\cdot\left( TAB-{CPX}_{2}+TSR+K_{D1}\cdot V_{C} \right)-\sqrt{\left( TAB-{CPX}_{2}+TSR+K_{D1}\cdot V_{C} \right)^{2}-4\cdot TSR\cdot\left( TAB-{CPX}_{2} \right)}$ (S16)

and similarly, for Eq. S15,

${CPX}_{2}= \frac{1}{2}\cdot\left( TAB-{CPX}_{1}+TCR+K_{D2}\cdot V_{C} \right)-\sqrt{\left( TAB-{CPX}_{1}+TCR+K_{D2}\cdot V_{C} \right)^{2}-4\cdot TCR\cdot\left( TAB-{CPX}_{1} \right)}$ (S17)

**Figure S1.**  Simulations from the TMDD model highlighting the relative concentrations of free monoclonal antibody (mAb) versus the mAb:cIL7R complex relative to the estimated concentration of cellular receptor (black dotted line) at a dose of 1 mg/kg q2w and 3 mg/kg q2w.

**
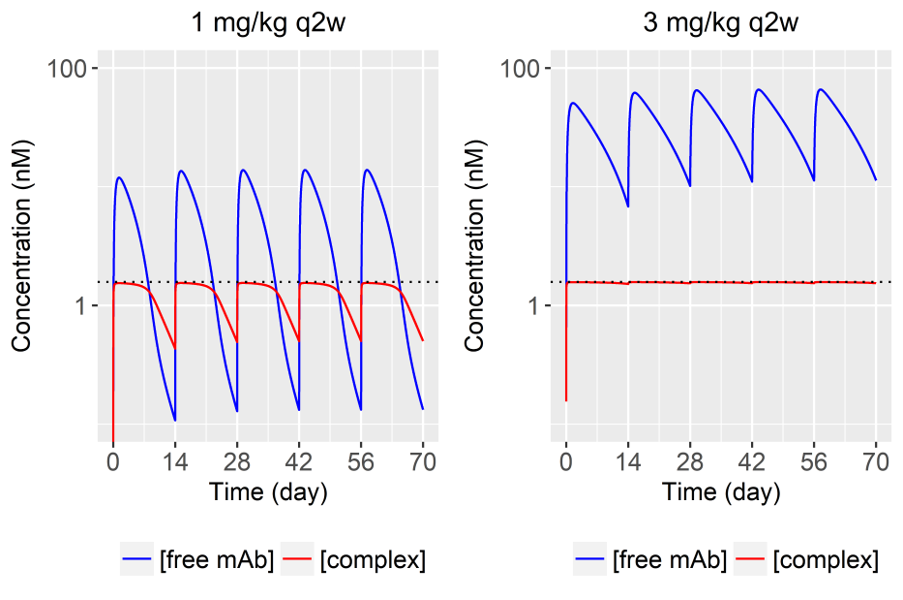
**

**Figure S2.** Visual predictive check (VPC) comparing the empirical 10^th^, 50^th^, and 90^th^ percentiles (red lines) with the simulated 10% (blue), 50% (pink) and 90% (blue) prediction intervals (PI) for percentage Free RO, stratified by total dose over 2 weeks.

**
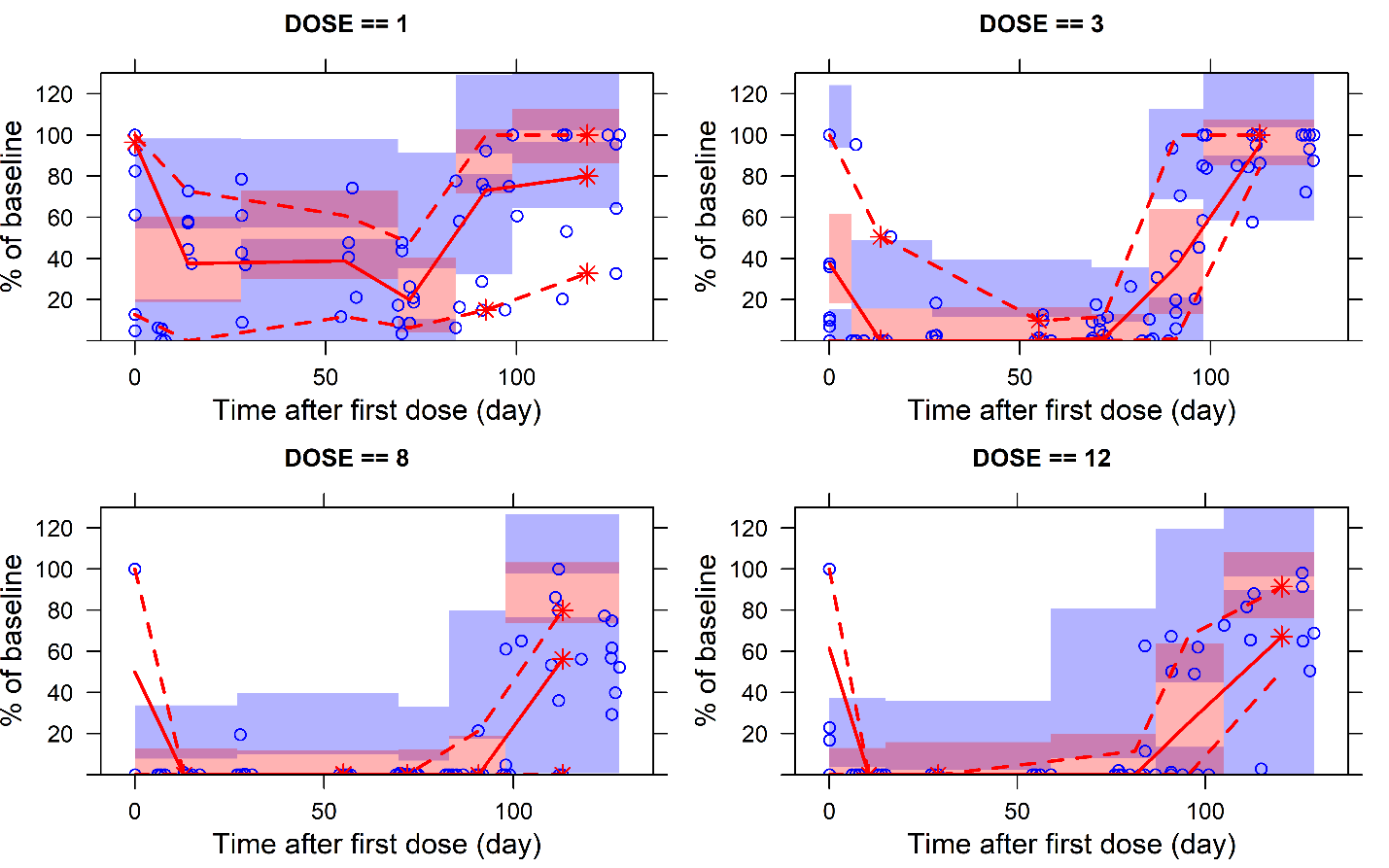
**

**Figure S3.** Individual model predictions and observations for PK.

**
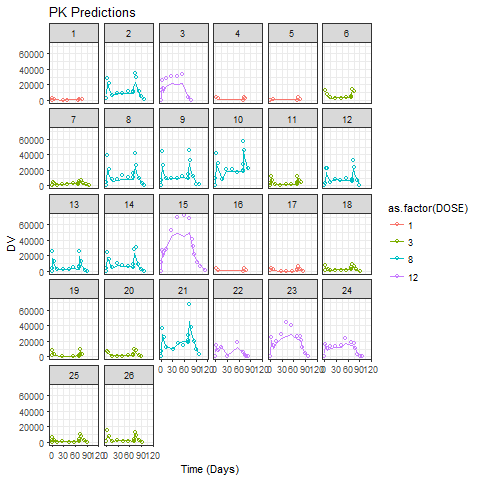
**

**Figure S4.** Individual model predictions and observations for sIL7Rα.

**
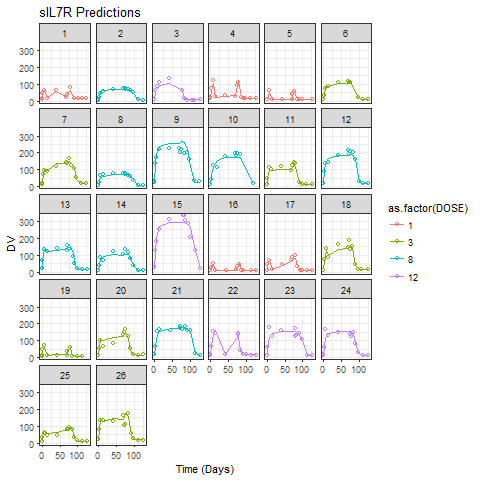
**

**Figure S5.** Individual model predictions and observations for Free RO.

**
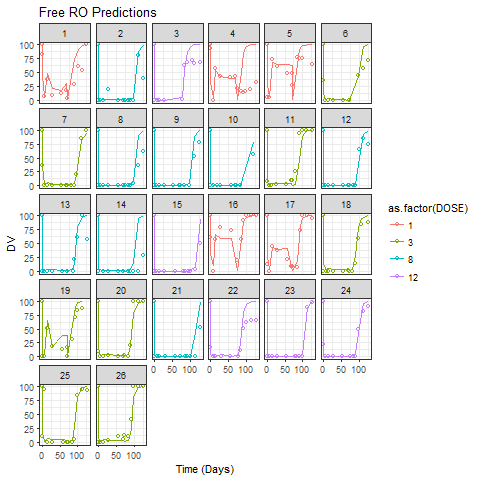
**

**Figure S6.** Individual model predictions and observations for CD4 EM T cells.

**
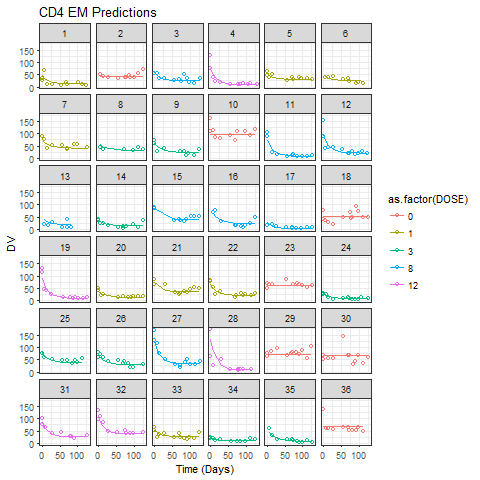
**

**Figure S7.** Individual model predictions and observations for CD4 Treg T cells.

**
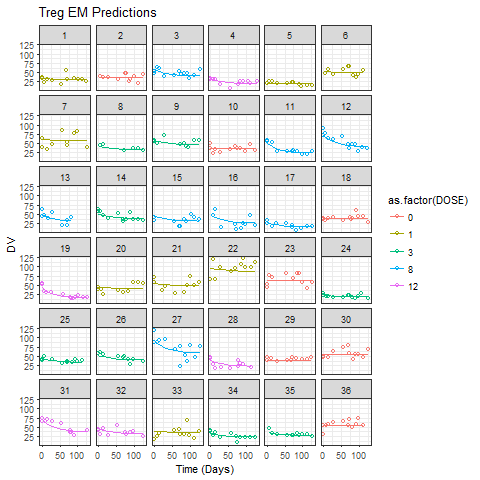
**

**Figure S8.** Goodness-of-fit diagnostics for PK.

**
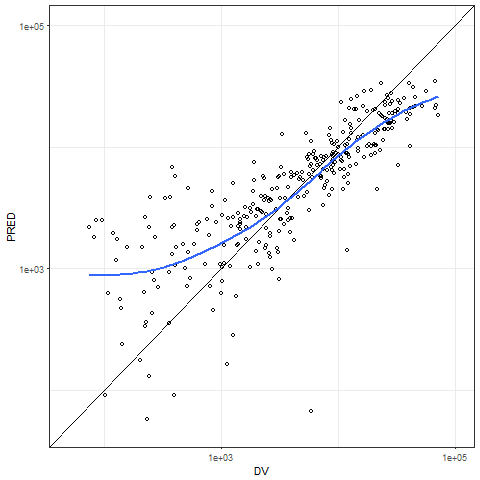

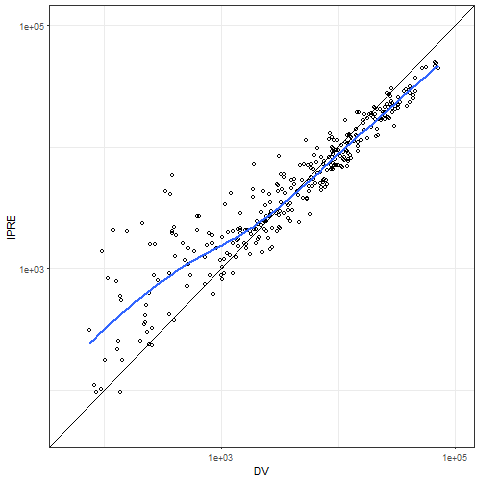
**

**
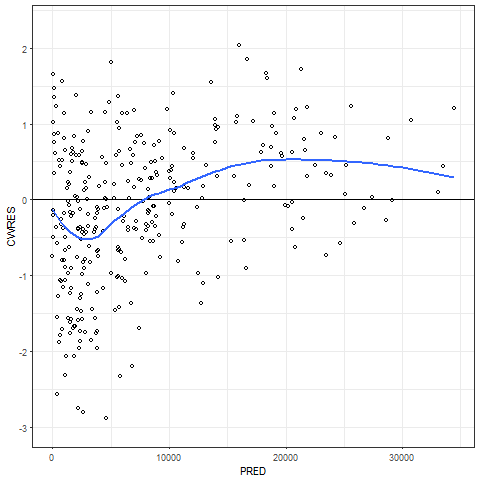

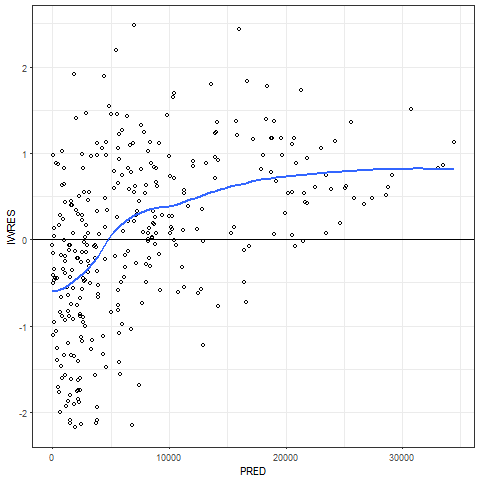
**

**
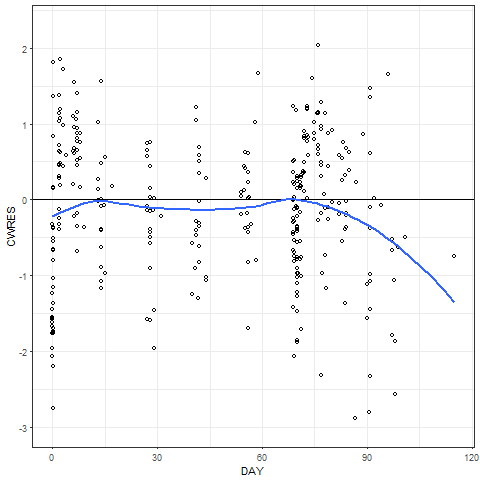

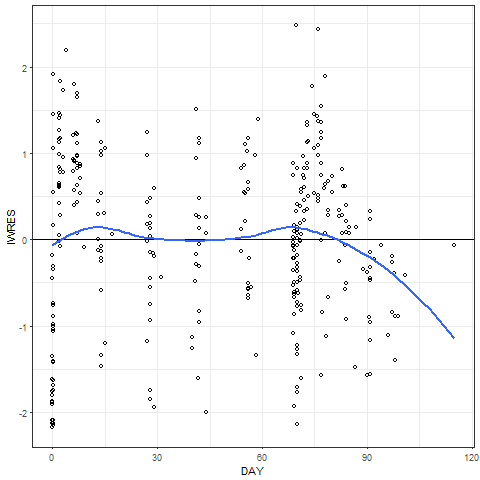
**

**Figure S9.** Goodness-of-fit diagnostics for sIL7Rα.


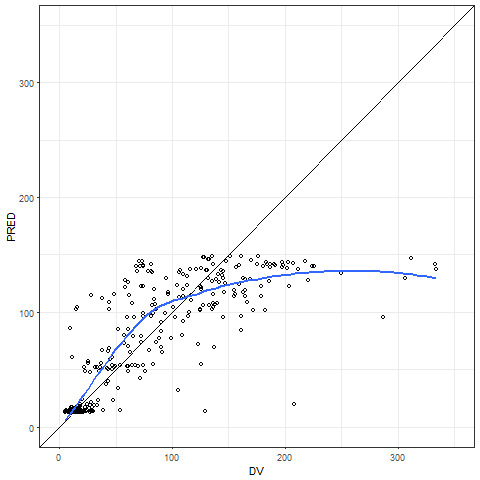

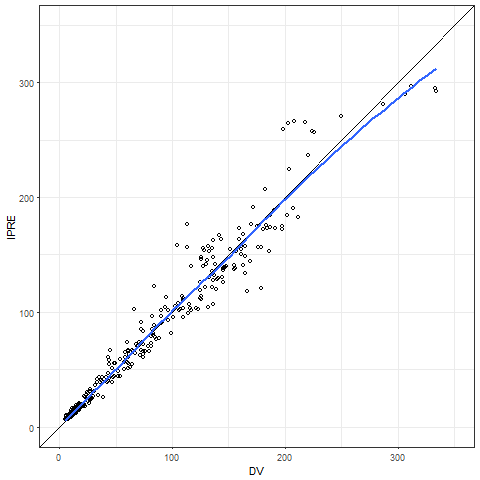


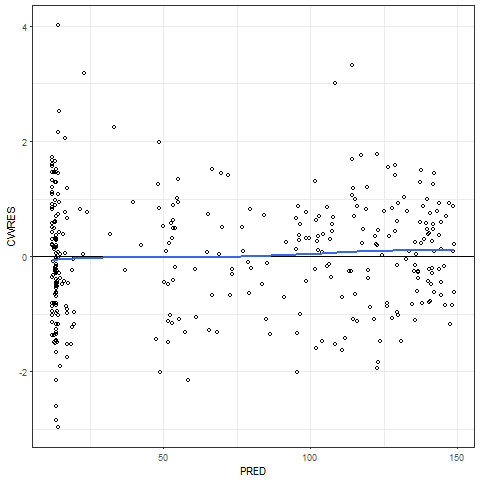

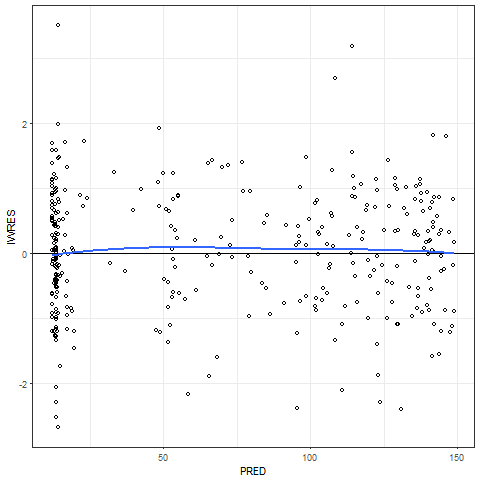


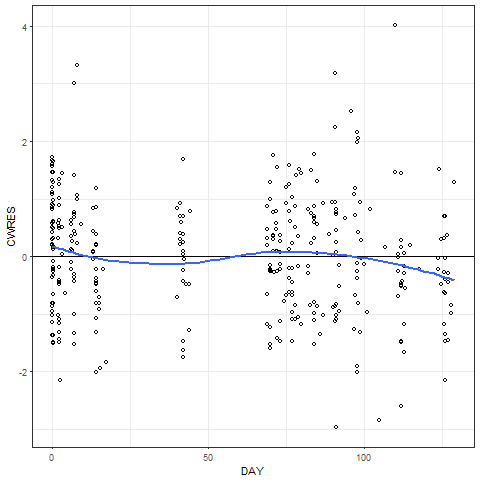

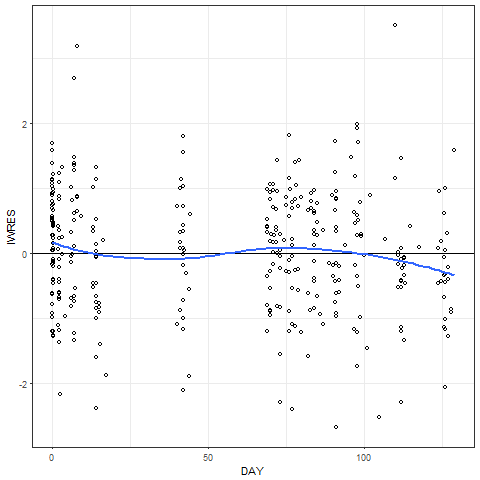


**Figure S10.** Goodness-of-fit diagnostics for Free RO.


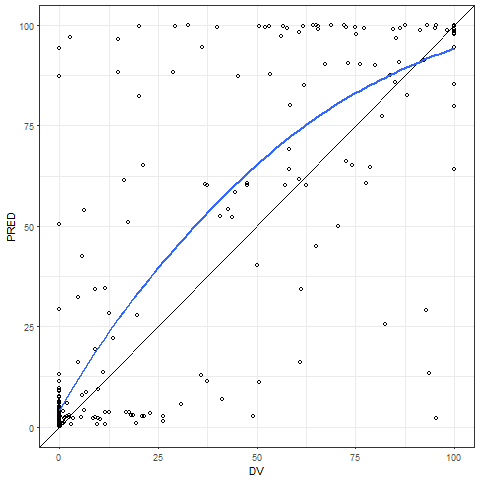

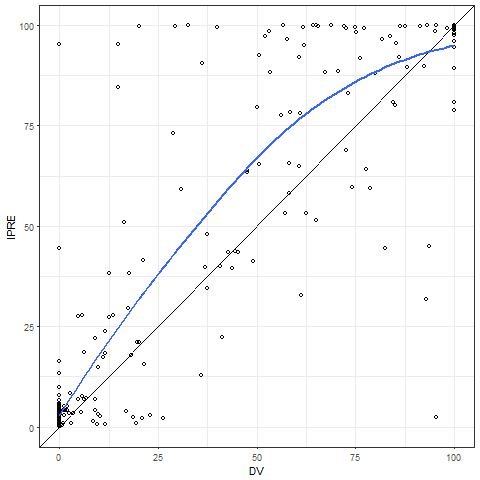


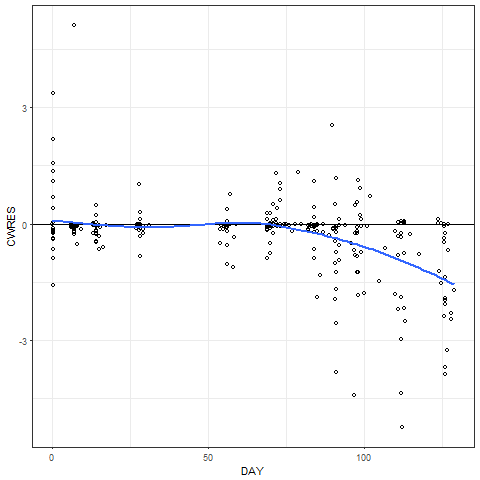

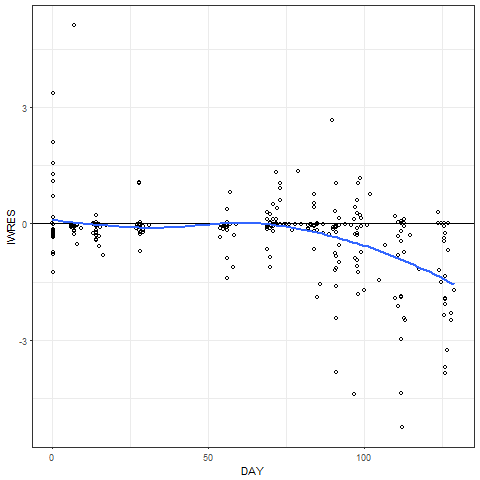


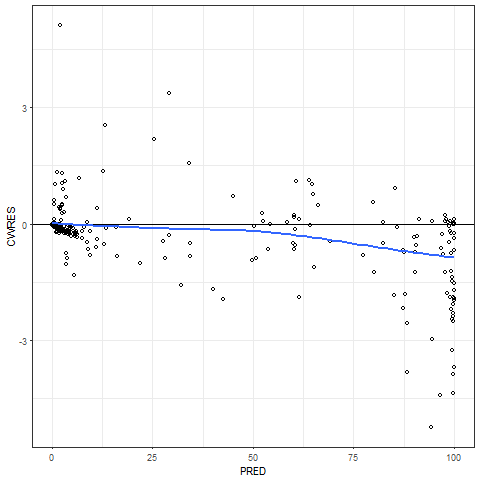

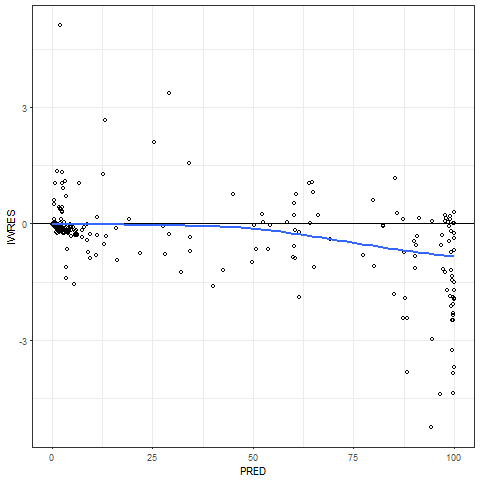


**Figure S11.** Goodness-of-fit diagnostics for CD4 EM T cells.


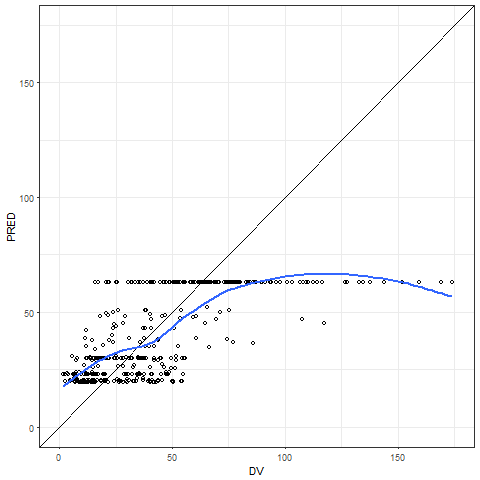

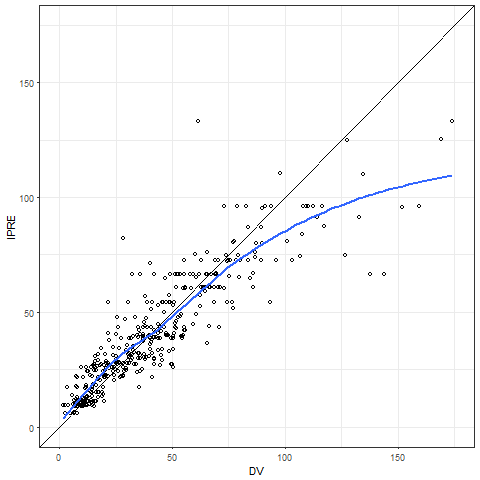


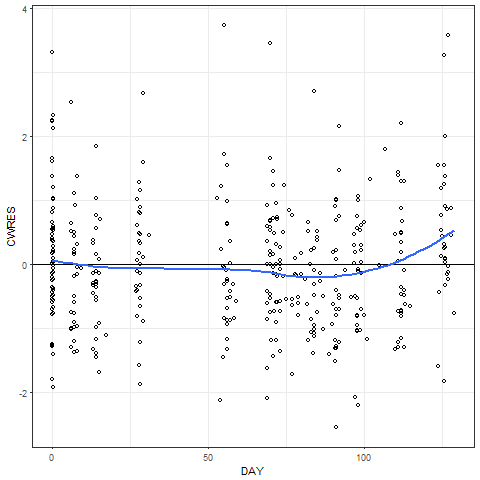

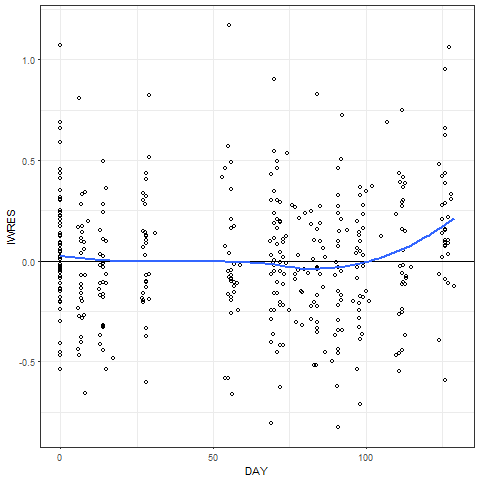


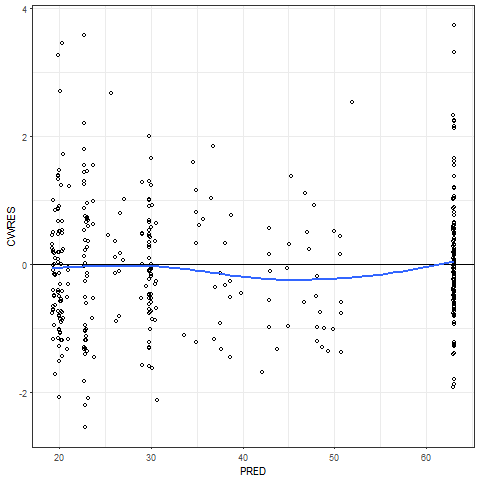

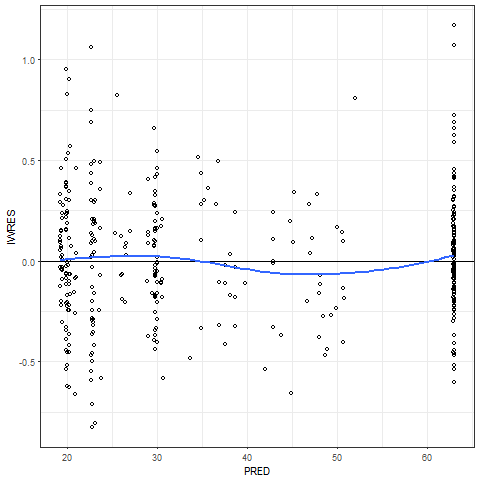


**Figure S12.** Goodness-of-fit diagnostics for CD4 Treg T cells.


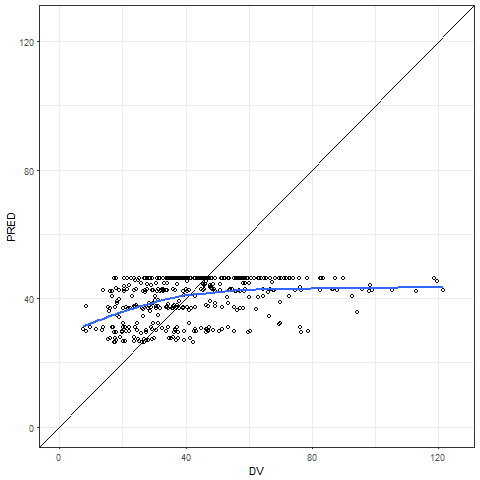

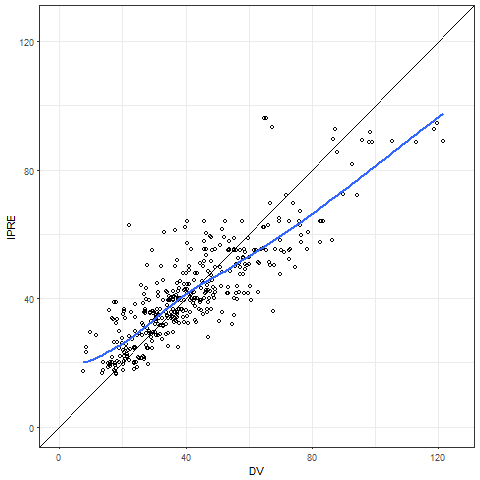


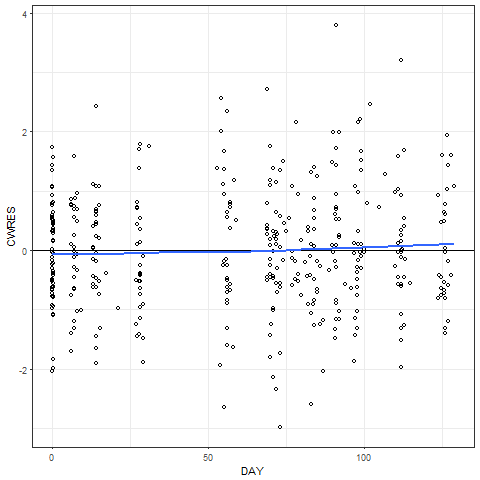

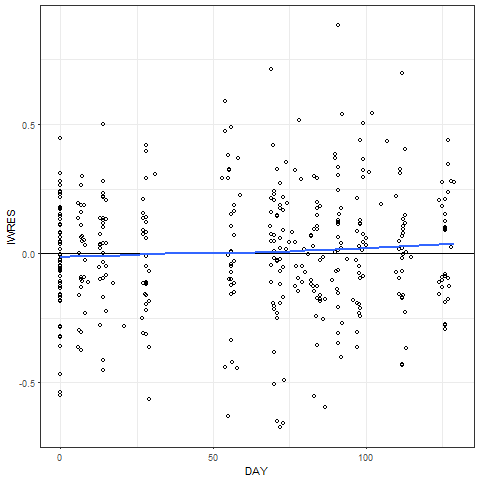


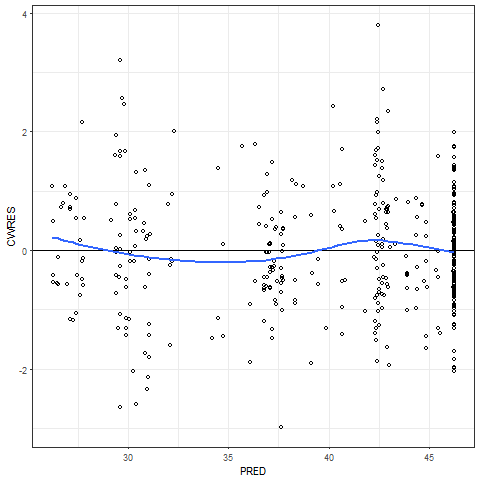

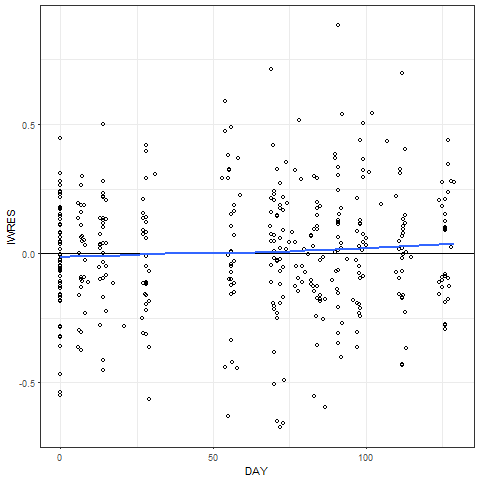


**NONMEM Code for Final Models**

$PROB run79.mod TMDD Model

$INPUT C ID VISI NTPD TAFD TIME DV AMT EVID DOSE

CMT DVID WT MDV COH ROUT RATE TYP SSID DROP DROP

$DATA B4351003_PKSLRO_28OCT2016_M1_TMDD_DEPO5.csv IGNORE=C

$SUBROUTINE ADVAN13 TOL=4

$MODEL

COMP =(TAB,DEFDOS) ; CMT1 is total antibody

COMP =(ABP) ; CMT2 is Ab second compartment

COMP =(TSR) ; CMT3 is total soluble receptor

COMP =(TCR) ; CMT4 is total cellular receptor

COMP =(SC) ; CMT5 is subcutaneous depot

$PK

D1 = 0.0833 ; 2 hour infusion

CLA = THETA(1)*EXP(ETA(1)) ; CL of antibody

VC = THETA(2)*EXP(ETA(2)) ; Central Volume

VP = THETA(3) ; Peripheral volume

Q2 = THETA(4) ; Distribution clearance

BIO = THETA(5) ; SC bioavailability

KA = THETA(6)*EXP(ETA(3)) ; SC absorption rate

CLSR = THETA(7) ; CL of soluble receptor

CLC1 = THETA(8) ; CL of RN168:sIL7R

KD1 = THETA(9) ; Binding Affinity RN168:sIL7R

BLSR = THETA(10)*EXP(ETA(4)) ;individual baseline (nM) sIL7R

KSY1 = BLSR*CLSR ; synthesis rate of soluble receptor

CLCR = THETA(11) ; CL of cellular receptor

CLC2 = CLCR ; CL of RN168:cIL7R complex

KD2 = THETA(12) ; Binding affinity RN168:cIL7R

BLCR = THETA(13) ; individual baseline (nM) cIL7R

KSY2 = BLCR*CLCR ; synthesis rate of cellular receptor

F1 = 1/0.15 ; Dose in mg(dataset), scaled to nmole

F5 = BIO*(1/0.15) ; Dose in mg(dataset), scaled to nmole x SC bioavail

A_0(1) = 0

A_0(2) = 0

A_0(3) = BLSR ; Initialize soluble receptor nM amount

A_0(4) = BLCR ; Initialize cellular receptor nM amount

$DES

TAB = A(1) ; total antibody central nmoles

ABP = A(2) ; free antibody in peripheral tissue

TSR = A(3) ; total soluble receptor nmoles

TCR = A(4) ; total cellular receptor nmoles

CPX1 = 0

CPX2 = 0

IF (TIME.GT.0) CPX2 = 0.5*((KD2*VC+TAB+TCR-CPX1)-SQRT((KD2*VC+TAB+TCR-CPX1)**2 -4*(TAB-CPX1)*TCR))

IF (TIME.GT.0) CPX1 = 0.5*((KD1*VC+TAB+TSR-CPX2)-SQRT((KD1*VC+TAB+TSR-CPX2)**2 -4*(TAB-CPX2)*TSR))

FAB = TAB-CPX1-CPX2 ; free antibody central

FSR = TSR-CPX1 ; free soluble receptor

FCR = TCR-CPX2 ; free cellular receptor

DADT(1)= KA*A(5) +Q2*(ABP/VP -FAB/VC) -(CLA/VC)*FAB -(CLC1/VC)*CPX1 -(CLC2/VC)*CPX2

DADT(2)= Q2*(FAB/VC -ABP/VP)

DADT(3)= KSY1 -(CLSR/VC)*FSR -(CLC1/VC)*CPX1

DADT(4)= KSY2 -(CLCR/VC)*FCR -(CLC2/VC)*CPX2

DADT(5)= -KA*A(5)

$ERROR

XTAB = A(1)

XPA1 = A(2)

XTSR = A(3)

XTCR = A(4)

XCX1 = 0

XCX2 = 0

IF (TIME.GT.0) XCX2 = 0.5*((KD2*VC+XTAB+XTCR-XCX1)-SQRT((KD2*VC+XTAB+XTCR-XCX1)**2 -4*(XTAB-XCX1)*XTCR))

IF (TIME.GT.0) XCX1 = 0.5*((KD1*VC+XTAB+XTSR-XCX2)-SQRT((KD1*VC+XTAB+XTSR-XCX2)**2 -4*(XTAB-XCX2)*XTSR))

CTAB = 150*(XTAB-XCX2)/VC ; measurable total antibody (ng/ml) (i.e. = Total - cIL7R bound)

CTSR = 30*A(3)/VC ; total sIL7R (ng/ml)

FREE = ((XTCR-XCX2)/XTCR)*100 ; % Free cIL7R

IF(DVID.EQ.1) THEN

IPRE=CTAB

IRES=DV-IPRE

W1 = THETA(14)

W2 = THETA(15)

W12 = SQRT(W1**2+(W2*IPRE)**2)

IWRE=IRES/W12

Y=IPRE+EPS(1)*W12

ENDIF

IF(FREE.LE.0) FREE=0

IF(DVID.EQ.2) THEN

IPRE=FREE

IRES=DV-IPRE

W3 = THETA(16)

W4 = THETA(17)

W34 = SQRT(W3**2+(W4*IPRE)**2)

IWRE=IRES/W34

Y=IPRE+EPS(1)*W34 ;combined

ENDIF

IF(DVID.EQ.3) THEN

IPRE=CTSR

IRES=DV-IPRE

W5 = THETA(18)

W6 = THETA(19)

W56 = SQRT(W5**2+(W6*IPRE)**2)

IWRE=IRES/W56

Y=IPRE+EPS(1)*W56 ;combined

ENDIF

$THETA

(0,0.382) ;1 [CLA]

(0,1.05) ;2 [VC]

(0,5) ;3 [VP]

1.1 FIX ;4 [Q2]

0.5 FIX ;5 [BIO]

(0,0.1) ;6 [KA]

(0,2.39) ;7 [CLSR]

(0,0.201 ) ;8 [CLC1]

(0,0.794 ) ;9 [KD1]

(0,0.29 ) ;10 [BLSR]

(0,9.67) ;11 [CLCR]

(0,0.395) ;12 [KD2]

(0,1) ;13 [BLCR]

0 FIX ;14 [add pk]

(0,0.103) ;15 [prop PK]

(0,17.5) ;16 [add RO]

0 FIX ;17 [prop RO]

0.01 FIX ;18 [add sIL7R]

(0,0.46) ;19 [prop sIL7R]

$OMEGA BLOCK(3)

0.1 ;IIV_CLA

0.01 0.1 ;IIV_VC

0.01 0.01 0.1 ;IIV_KA

$OMEGA

0.1 ;IIV_BLSR

$SIGMA

1 FIX

$EST METHOD=1 INT MAXEVAL=9999 PRINT=5 NOABORT FILE=run79.ext

SIGL=3 NSIG=1 NOTHETABOUNDTEST NOOMEGABOUNDTEST NOSIGMABOUNDTEST

$COVARIANCE PRINT=E UNCONDITIONAL SIGL=12 TOL=12

$TABLE ID SSID DVID TIME IPRE IWRE DOSE NOPRINT ONEHEADER FORMAT=s1PE13.7 FILE=sdtab79

$TABLE ID CLA VC VP Q2 KA KD1 CLSR CLC1 BLSR CLCR ETA1 ETA2

ETA3 ETA4 NOPRINT ONEHEADER FILE=patab79

$TABLE ID WT NOPRINT ONEHEADER FILE=cotab79

$TABLE ID COH DVID NOPRINT ONEHEADER FILE=catab79

$PROBLEM run33.mod TREG Model

$INPUT ID DVID DV DOSE DAY SSID

$DATA B4351003_EM_TREG_31OCT2018.csv ACCEPT=(DVID.EQ.5) IGNORE=@

$PRED

EMAX = THETA(1)*EXP(ETA(1))

ED50 = THETA(2)*EXP(ETA(2))

BL = THETA(3)*EXP(ETA(3))

KOUT = THETA(4)*EXP(ETA(4))

EFF = 1-(EMAX*DOSE/(ED50+DOSE))

IPRE = BL*(EXP(-KOUT*DAY)+EFF*(1-EXP(-KOUT*DAY)))

W = IPRE

IRES = DV-IPRE

IWRES = IRES/W

Y = IPRE + IPRE*EPS(1) + EPS(2)

$THETA

(0,0.7,1) ;EMAX

(0,0.2,10) ; ED50

(0,50) ; BL

(0,0.05) ; KOUT

$OMEGA

0.02

0 FIX

0.02

0 FIX

$SIGMA

0.01

0 FIX

$EST METHOD=1 INT MAXEVAL=9999 PRINT=5 NOABORT FILE=run33.ext

$COVARIANCE PRINT=E UNCONDITIONAL

$TABLE ID SSID DVID DAY IPRE IWRES CWRES DOSE NOPRINT ONEHEADER FORMAT=s1PE13.7 FILE=sdtab33

$TABLE ID EMAX ED50 KOUT BL ETA1 ETA3 ETA4 NOPRINT ONEHEADER FILE=patab33

$TABLE ID NOPRINT ONEHEADER FILE=cotab33

$TABLE ID DOSE DVID NOPRINT ONEHEADER FILE=catab33

$PROBLEM run76.mod EM model

$INPUT ID DVID DV DOSE DAY SSID

$DATA B4351003_EM_TREG_31OCT2018.csv ACCEPT=(DVID.EQ.4) IGNORE=@

$PRED

EMAX = THETA(1)*EXP(ETA(1))

ED50 = THETA(2)*EXP(ETA(2))

BL = THETA(3)*EXP(ETA(3))

KOUT = THETA(4)*EXP(ETA(4))

EFF = 1-(EMAX*DOSE/(ED50+DOSE))

IPRE = BL*(EXP(-KOUT*DAY)+EFF*(1-EXP(-KOUT*DAY)))

W = IPRE

IRES = DV-IPRE

IWRES = IRES/W

Y = IPRE + IPRE*EPS(1) + EPS(2)

$THETA

(0,0.7,1) ;EMAX

(0,0.5,10) ; ED50

(0,50) ; BL

(0,0.05) ; KOUT

$OMEGA

0.02

0 FIX

0.02

0 FIX

$SIGMA

0.01

0 FIX

$EST METHOD=1 INT MAXEVAL=9999 PRINT=5 NOABORT FILE=run76.ext

$COVARIANCE PRINT=E UNCONDITIONAL

$TABLE ID SSID DVID DAY IPRE IWRES CWRES DOSE NOPRINT ONEHEADER FORMAT=s1PE13.7 FILE=sdtab76

$TABLE ID EMAX ED50 KOUT BL ETA1 ETA3 ETA4 NOPRINT ONEHEADER FILE=patab76

$TABLE ID NOPRINT ONEHEADER FILE=cotab76

$TABLE ID DOSE DVID NOPRINT ONEHEADER FILE=catab76

**Simulation Code**

## load libraries

library(ggplot2)

library(dplyr)

library(tidyr)

library(mrgsolve)

#################### model ################################

code <- '

$PARAM CLA=0.933, VC=1.07, Q=1.1, VP=4.96, BIO=0.5, KA=0.209, CLSR=2.45, CLC1=0.197,

KD1=1.01, BLSR=0.45, CLCR=9.39, KD2=0.285, BLCR=1.58, KIN1=4.196, KOUT1=0.0665, EMAX1=0.715,

ED501=0.353, KIN2=1.42, KOUT2=0.0308, EMAX2=0.7, ED502=7.06, DOSE=1

$CMT TAB, ABP, TSR, TCR, SC, EM, TREG

$SET delta=72

$MAIN

_F(1) = 1/0.15;

_F(5) = BIO*(1/0.15);

double BLEM=KIN1/KOUT1;

double BLTR=KIN2/KOUT2;

double CLC2=CLCR;

TSR_0 = BLSR;

TCR_0 = BLCR;

EM_0 = BLEM;

TREG_0 = BLTR;

double KSY1=BLSR*CLSR;

double KSY2=BLCR*CLCR;

$ODE

double CPX1 = 0.5*((KD1*VC+TAB+TSR-CPX2)-sqrt(pow(KD1*VC+TAB+TSR-CPX2,2) -4*(TAB-CPX2)*TSR));

double CPX2 = 0.5*((KD2*VC+TAB+TCR-CPX1)-sqrt(pow(KD2*VC+TAB+TCR-CPX1,2) -4*(TAB-CPX1)*TCR));

double FAB = TAB-CPX1-CPX2;

double FSR = TSR-CPX1;

double FCR = TCR-CPX2;

double EFFECT1= 1-(EMAX1*DOSE/(ED501+DOSE));

double EFFECT2= 1-(EMAX2*DOSE/(ED502+DOSE));

dxdt_TAB = KA*SC +Q*(ABP/VP -FAB/VC) -(CLA/VC)*FAB -(CLC1/VC)*CPX1 -(CLC2/VC)*CPX2;

dxdt_ABP = Q*(FAB/VC -ABP/VP);

dxdt_TSR = KSY1 -(CLSR/VC)*FSR -(CLC1/VC)*CPX1;

dxdt_TCR = KSY2 -(CLCR/VC)*FCR -(CLC2/VC)*CPX2;

dxdt_SC = -KA*SC;

dxdt_EM = KIN1*EFFECT1 - KOUT1*EM ;

dxdt_TREG = KIN2*EFFECT2 - KOUT2*TREG;

$TABLE

double CTAB = 150*(TAB-CPX2)/VC;

double CFAB = 150*(TAB-CPX1-CPX2)/VC;

double CTSR = 30*TSR/VC;

double FREE = ((TCR-CPX2)/TCR)*100;

double PCB_EM = ((EM-BLEM)/BLEM)*100;

double PCB_TREG = ((TREG-BLTR)/BLTR)*100;

double CCPX2NM = CPX2/VC;

double CCPX1NM = CPX1/VC;

double CTCRNM = TCR/VC;

double CTSRNM = TSR/VC;

double CTABNM = (TAB-CPX2)/VC;

double CFABNM = FAB/VC;

double CFCRNM = FCR/VC;

double RATIO1 = CTABNM/CCPX2NM;

double RATIO2 = CTCRNM/CCPX2NM;

$CAPTURE EM TREG CTAB CFAB CTSR FREE PCB_EM

PCB_TREG CTCRNM CTSRNM CTABNM CCPX2NM CCPX1NM CFABNM CFCRNM RATIO1 RATIO2

'

#######################################################################

# COMPILE MODELS

#######################################################################

dir<-tempdir()

mod <- mread(code=code, model="pkpd",proj=dir) #

#######################################################################

# SIMULATIONS ##

#######################################################################

dose <- c(1,3,6)

wt <- 70

mod <- mod %>% param(CLA=0.999, VC=1.1, VP=5.28,Q=1.1,BIO=0.5,KA=0.211,CLSR=2.24,

CLC1=0.196,KD1=0.779,BLSR=0.45,CLCR=10.4,KD2=0.450,BLCR=1.37)

data<-data.frame(ID=1, evid=1, time=0, cmt=5,amt=1*wt,addl=5,ii=14,dose=1)

sim1<- mod %>% data_set(data) %>% carry.out(evid,time,amt,dose,CTAB) %>% update(start=0,end=70,delta=0.1) %>% mrgsim

sim1<-as.data.frame(sim1)

sim1<-subset(sim1, evid==0)

mod1 <- mod %>% param(DOSE=3)

data<-data.frame(ID=2, evid=1, time=0, cmt=5,amt=3*wt,addl=5,ii=14,dose=3)

sim2<- mod1 %>% data_set(data) %>% carry.out(evid,time,amt,dose) %>% update(start=0,end=70,delta=0.1) %>% mrgsim

sim2<-as.data.frame(sim2)

sim2<-subset(sim2, evid==0)

mod1 <- mod %>% param(DOSE=6)

data<-data.frame(ID=3, evid=1, time=0, cmt=5,amt=6*wt,addl=5,ii=14,dose=6)

sim3<- mod1 %>% data_set(data) %>% carry.out(evid,time,amt,dose) %>% update(start=0,end=70,delta=0.1) %>% mrgsim

sim3<-as.data.frame(sim3)

sim3<-subset(sim3, evid==0)

sim4 <- rbind(sim1,sim2,sim3)

#PK#

ggplot(sim4,aes(x=time,y=CTAB,color=as.factor(ID)))+

geom_line()+

scale_y_log10(limits=c(0.1,100000))+

scale_x_continuous(breaks=c(0,14,28,42,56,70,84))+

#geom_line(data=sim,aes(x=time,y=CFAB),linetype="dashed")+

ggtitle("PF-06342674")+

labs(y='Concentration (ng/mL)', x="Time (day)")+

theme_bw()+

theme(legend.justification=c(1,0), legend.position=c(1,0))+

theme(

axis.text = element_text(size = 14),

axis.title= element_text(size = 14),

legend.text=element_text(size=14))+

theme(plot.title = element_text(hjust = 0.5))+

theme(legend.position="none")+

scale_color_manual(name="Q2W Dose",

values=c("red", "darkgreen","blue"),

breaks=c(1,2,3),

labels=c("1 mg/kg", "3 mg/kg","6 mg/kg"))

#sIL7R#

ggplot(sim4,aes(x=time,y=CTSR,color=as.factor(ID)))+

geom_line()+

#scale_y_continuous(limits = c(0, 100))+

scale_x_continuous(breaks=c(0,14,28,42,56,70,84))+

ggtitle(expression(paste("sIL7R",alpha)))+

labs(y='Concentration (ng/mL)', x="Time (day)")+

theme_bw()+

theme(legend.justification=c(1,0), legend.position=c(1,0))+

theme(plot.title = element_text(hjust = 0.5))+

theme(

axis.text = element_text(size = 14),

axis.title= element_text(size = 14),

legend.text=element_text(size=14))+

theme(legend.position="none")+

scale_color_manual(name="Q2W Dose",

values=c("red", "darkgreen","blue"),

breaks=c(1,2,3),

labels=c("1 mg/kg", "3 mg/kg","6 mg/kg"))

#Free RO#

ggplot(sim4,aes(x=time,y=FREE,color=as.factor(ID)))+

geom_line()+

#scale_y_continuous(limits = c(0, 100))+

scale_x_continuous(breaks=c(0,14,28,42,56,70,84))+

ggtitle("Free RO")+

labs(y='Free Receptor Occupancy (%)', x="Time (day)")+

theme_bw()+

theme(legend.justification=c(1,1), legend.position=c(1,1))+

theme(plot.title = element_text(hjust = 0.5))+

theme(

axis.text = element_text(size = 14),

axis.title= element_text(size = 14),

legend.text=element_text(size=14))+

theme(legend.position="none")+

scale_color_manual(name="Q2W Dose",

values=c("red", "darkgreen","blue"),

breaks=c(1,2,3),

labels=c("1 mg/kg", "3 mg/kg","6 mg/kg"))

#EM/TREG#

ggplot(sim4,aes(x=time,y=PCB_EM,color=as.factor(ID)))+

geom_line()+

geom_line(data=sim4,aes(x=time,y=PCB_TREG),linetype="dashed")+

scale_y_continuous(limits = c(-100, 0))+

scale_x_continuous(breaks=c(0,14,28,42,56,70,84))+

ggtitle("EM and Treg")+

labs(y='Percent change from baseline (%)', x="Time (day)")+

theme_bw()+

theme(legend.justification=c(1,0), legend.position=c(1,0))+

theme(plot.title = element_text(hjust = 0.5))+

theme(

axis.text = element_text(size = 14),

axis.title= element_text(size = 14),

legend.text=element_text(size=14))+

theme(legend.position="none")+

scale_color_manual(name="Q2W Dose",

values=c("red", "darkgreen","blue"),

breaks=c(1,2,3),

labels=c("1 mg/kg", "3 mg/kg","6 mg/kg"))

#Treg/EM ratio#

sim4$ratio <- sim4$TREG/sim4$EM

ggplot(sim4,aes(x=time,y=ratio,color=as.factor(ID)))+

geom_line()+

#scale_y_continuous(limits = c(0, -80))+

scale_x_continuous(breaks=c(0,14,28,42,56,70,84))+

ggtitle("Treg/EM ratio")+

labs(y='Absolute count Treg/EM Ratio', x="Time (day)")+

theme_bw()+

theme(legend.justification=c(1,0), legend.position=c(1,0))+

theme(plot.title = element_text(hjust = 0.5))+

theme(axis.text = element_text(size = 14),

axis.title= element_text(size = 14),

legend.text=element_text(size=14))+

theme(legend.position="none")+

scale_color_manual(name="Q2W Dose",

values=c("red", "darkgreen","blue"),

breaks=c(1,2,3),

labels=c("1 mg/kg", "3 mg/kg","6 mg/kg"))
